# PKD autoinhibition in *trans* regulates activation loop autophosphorylation in *cis*

**DOI:** 10.1073/pnas.2212909120

**Published:** 2023-02-06

**Authors:** Ronja Reinhardt, Kai Hirzel, Gisela Link, Stephan A. Eisler, Tanja Hägele, Matthew A. H. Parson, John E. Burke, Angelika Hausser, Thomas A. Leonard

**Affiliations:** ^a^Department of Structural and Computational Biology, Max Perutz Labs, Campus Vienna Biocenter, Vienna 1030, Austria; ^b^Department of Medical Biochemistry, Medical University of Vienna, Vienna 1090, Austria; ^c^Institute of Cell Biology and Immunology, University of Stuttgart 70569, Stuttgart, Germany; ^d^Stuttgart Research Center Systems Biology, University of Stuttgart 70569, Stuttgart, Germany; ^e^Department of Biochemistry and Microbiology, University of Victoria, Victoria, BC, Canada V8W 2Y2; ^f^Department of Biochemistry and Molecular Biology, The University of British Columbia, Vancouver BC V6T 1Z3, Canada

**Keywords:** autoinhibition, autophosphorylation, kinase, *cis*, *trans*

## Abstract

Phosphorylation is a ubiquitous mechanism by which signals are transduced in cells. Protein kinases, enzymes that catalyze the phosphotransfer reaction are, themselves, often regulated by phosphorylation. Paradoxically, however, a substantial fraction of more than 500 human protein kinases are capable of catalyzing their own activation loop phosphorylation. Commonly, these kinases perform this autophosphorylation reaction in *trans*, whereby transient dimerization leads to the mutual phosphorylation of the activation loop of the opposing protomer. In this study, we demonstrate that protein kinase D (PKD) is regulated by the inverse mechanism of dimerization-mediated *trans*-autoinhibition, followed by activation loop autophosphorylation in *cis*. We show that PKD forms a stable face-to-face homodimer that is incapable of either autophosphorylation or substrate phosphorylation. Dissociation of this *trans*-autoinhibited dimer results in activation loop autophosphorylation, which occurs exclusively in *cis*. Phosphorylation serves to increase PKD activity and prevent *trans*-autoinhibition, thereby switching PKD on. Our findings not only reveal the mechanism of PKD regulation but also have profound implications for the regulation of many other eukaryotic kinases.

## Significance

Many eukaryotic protein kinases are activated by phosphorylation of their activation loop. Some protein kinases can autoactivate by phosphorylating their own activation loop. This reaction is widely believed to occur exclusively in *trans* (by a copy of the same protein) and has been observed in a wide array of kinases that undergo stimulus-induced dimerization. Here, we describe the inverse mechanism for activation of protein kinase D (PKD), an essential kinase involved in membrane trafficking. We show that inactive PKD is dimeric and that its activation depends on dissociation of its kinase domains followed by activation loop autophosphorylation in *cis* (by itself). Nature has, therefore, found two solutions to the same problem that are simply the inverse of one another.

Protein phosphorylation is the most abundant and important posttranslational modification in cellular signal transduction (1). Hence, protein kinases are versatile switches in countless signaling cascades, and their dysregulation is a major threat to the fidelity of cell signaling and, eventually, the health of an organism. Protein kinases are, therefore, tightly regulated enzymes that integrate various signaling inputs to drive downstream signaling in the form of substrate phosphorylation. While the active conformation of a kinase domain is conserved among the entire class of enzymes, the inactive conformations that are used to suppress their activity in the absence of a physiological stimulus are diverse (2). Importantly, the inactive state of a kinase is not just the absence of its active conformation but well-defined and tightly regulated conformations that rely on a limited repertoire of reoccurring patterns (3). As such, the inactive conformation can represent a unique and highly specific target for pharmacological intervention (4), while inhibitors that target the highly conserved active conformation are prone to off-target effects (5).

A major determinant for the activity status of a protein kinase is the trajectory of a flexible loop within the kinase called the activation loop. While its inactive conformation can vary greatly (3), active kinases, in contrast, adopt a single, well-defined conformation of their activation loop (6). The acquisition of the active conformation is, in many eukaryotic protein kinases, accomplished through phosphorylation of a conserved serine, threonine, or tyrosine residue in the activation loop by an upstream kinase. Phosphorylation creates a network of hydrogen bonds and electrostatic interactions that stabilizes the packing of an otherwise labile loop against the kinase domain, which both creates the surface for substrate binding and organizes the catalytic machinery for productive phosphotransfer (6, 7).

A special case is kinases that phosphorylate their own activation loop. While more than 18% of protein kinases have been reported to autophosphorylate their activation loop (8), the mechanisms by which they acquire autocatalytic activity are still debated. Multiple models have been proposed that cover activation loop autophosphorylation in both *cis* and *trans* (8, 9), although the majority of kinases that autophosphorylate are believed to do so in *trans* (8). Logic implies that these kinases possess an intrinsic capacity to autophosphorylate in the absence of phosphorylation of their own activation loop. Therefore, the autophosphorylation reaction is necessarily mechanistically distinct from the classical mode of substrate phosphorylation.

**Fig. 1.**
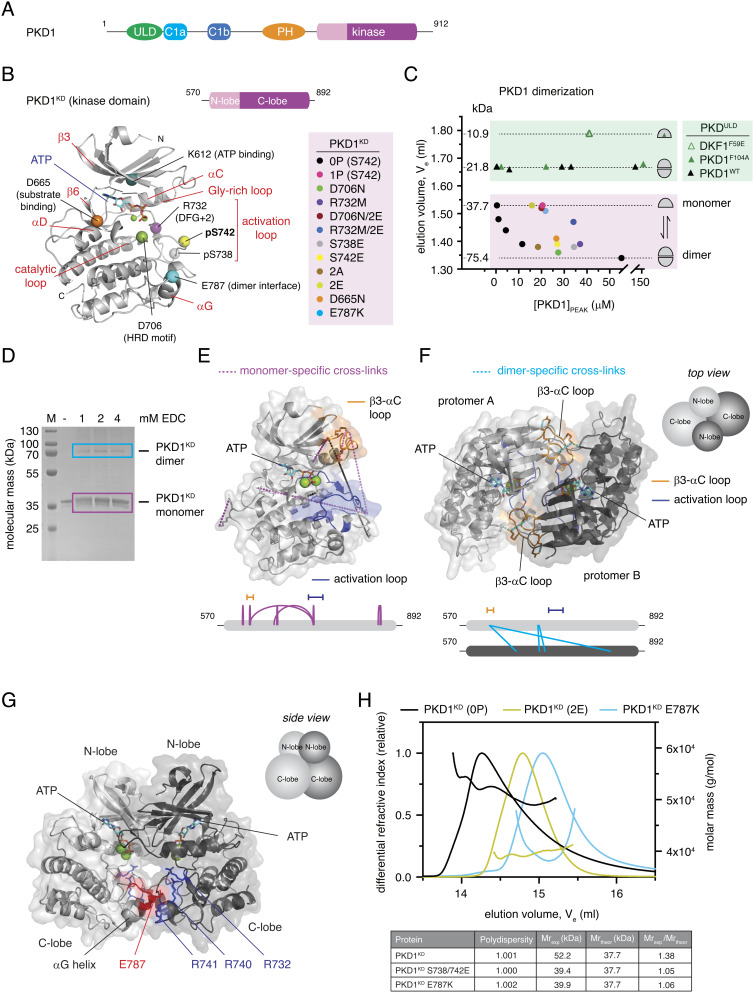
The PKD kinase domain forms a face-to-face dimer. (*A*) Domain architecture of human PKD1. (*B*) PKD variants (color-coded) employed in this study are mapped onto a homology model of the PKD1 kinase domain (PKD1^KD^). (*C*) Analytical size exclusion chromatography of PKD1^ULD^ and PKD1^KD^ variants. Dashed lines correspond to monomer and dimer calibrated by multiangle light scattering to determine absolute molecular mass. (*D*) Cross-linking of PKD1^KD^ with 1-ethyl-3-(3-dimethylaminopropyl)carbodiimide (EDC). Monomer (magenta box) and dimer (cyan box) bands were subjected to mass spectrometry analysis. (*E*) Monomer-specific cross-links mapped onto the Rosetta homology model of PKD1^KD^. Activation loop (blue) and β3-αC loop (orange) are indicated. *Below*: schematic of EDC cross-links specific to the monomer band (magenta box in *D*). (*F*) Dimer-specific cross-links mapped onto the cross-link–constrained model of the PKD1^KD^ dimer obtained from comparative modeling in Rosetta with C2 symmetry. *Below*: schematic of EDC cross-links specific to the dimer band (cyan box in *D*). (*G*) *Side* view of the experimentally constrained dimer model of PKD1^KD^. The conserved acidic patch at the tip of the αG-helix (red) and conserved basic residues in the activation loop (blue) are indicated. (*H*) Size exclusion chromatography coupled to multiangle light scattering (SEC–MALS) of wild-type (black), S738E/S742E (yellow green), and E787K (cyan) variants of PKD1^KD^.

One example of a kinase reported to autophosphorylate is protein kinase D (PKD). PKD is a family of Ser/Thr kinases of the calcium/calmodulin-dependent kinase (CAMK) family comprising three isoforms, PKD1, 2, and 3, which all share the same domain arrangement (Fig. 1*A*). In epithelial cells, PKD is located at the *trans*-Golgi network (TGN), where its activity is required for the formation of secretory cargo carriers of the TGN to the cell surface (CARTS) (10, 11). PKD activation depends on binding of its C1 domains to diacylglycerol (DAG) in the TGN (12, 13), which results in its autophosphorylation on S742 and activation (14, 15). A secondary, noncanonical, phosphorylation site (S738) in the activation loop of PKD1 has been reported (14, 16), although its physiological relevance is not clear. Once activated, PKD regulates vesicular transport by phosphorylating and activating the lipid kinase PI4KIIIβ (17) among other substrates (18, 19). In previous work, we have shown that activation loop phosphorylation of PKD is dependent on a ubiquitin-like dimerization domain (ULD) in its N terminus (15). However, the structural mechanism underlying phosphorylation-mediated PKD activation remained elusive.

In this study, we biochemically dissect the mechanisms of autoinhibition and activation of PKD. We show that the kinase domain of PKD, counterintuitively, forms an inactive dimer. We demonstrate that activation loop autophosphorylation is promoted by dissociation of the inactive dimer and that phosphorylation both increases kinase activity and prevents reassociation of the kinase domain. We present biochemical evidence that autophosphorylation and substrate phosphorylation are mechanistically distinct and that activation loop autophosphorylation occurs, not in *trans* but in *cis*. Finally, we show that dissociation of the kinase domains of inactive PKD dimers and their subsequent activation loop autophosphorylation is essential for constitutive secretion in cells.

## Results

### PKD Kinase Domain Forms a Face-to-Face Dimer.

PKD activation and autophosphorylation in cells are dependent on the formation of a homodimer that is mediated via its N-terminal ULD and C-terminal kinase domain. The unphosphorylated kinase domain of PKD forms a stable dimer in solution, while activation loop phosphorylation prevents its dimerization (15), but the mechanism by which phosphorylation activates PKD is, as yet, unknown. To obtain structural insights into the dimerization of the kinase domain of PKD, we built in silico models of both the monomeric and dimeric states of the kinase domain guided by a combination of careful site-directed mutagenesis (Fig. 1*B*), oligomeric state determination (Fig. 1*C*), in-solution cross-linking (Fig. 1*D*), and evolutionary sequence conservation.

All recombinant proteins used in this study were purified to homogeneity and subsequently analyzed by intact mass spectrometry to confirm their purity and precise chemical identity (*SI Appendix*, Figs. S1 and S2). The PKD1 kinase domain construct, henceforth referred to as PKD1^KD^, comprises residues 570 to 890 and includes, in addition to the canonical kinase domain, short flanking regions with high sequence conservation in vertebrate PKD orthologs. The oligomeric state of each protein was evaluated by analytical size exclusion chromatography (Fig. 1*C*), for which the correlation of elution volumes to molecular masses was calibrated by size exclusion chromatography coupled to multiangle light scattering (SEC–MALS).

We first used the comparative modeling tool of the Rosetta in silico modeling suite (20) to predict the structure of the PKD1 kinase domain based on published crystal structures of the related protein kinases Chk2 (pdb: 3i6U), DAPK2 (pdb: 2a2a, 2yaa), and phosphorylase kinase (pdb: 2phk), taking into account the correct geometry and stereochemistry of ATP and Mg^2+^ binding (Fig. 1*E*). Comparison of the resulting model to the AlphaFold prediction (AF-Q15139-F1) (21) shows strong agreement, with an overall RMSD of 0.680 Å over all C_α_ atoms (*SI Appendix*, Fig. S3*A*).

To provide experimental restraints for in silico modeling of the dimeric state, we performed in-solution cross-linking with the heterobifunctional, zero-length cross-linker 1-ethyl-3-(3-dimethylaminopropyl)carbodiimide hydrochloride (EDC) on dephosphorylated PKD1^KD^ (Fig. 1*D*). Since thermal stability measurements of the PKD kinase domain indicated the strongest stabilization by ATP (*SI Appendix*, Fig. S3*B*), cross-linking was performed under conditions of saturating ATP and MgCl_2_. Mass spectrometric analysis of cross-linked residues in both the monomeric and the dimeric species isolated from the gel (Fig. 1*D*) revealed several major changes upon dimerization (Fig. 1 *E* and *F*, and *SI Appendix*, Table S1). Cross-links from the activation loop residues K737 to E668 in the αD-helix and E624 in the β3-αC loop were unique to the monomer, while cross-linking of E736 in the activation loop and K622 in the β3-αC loop was reduced in the dimer. Together with an abundance of monomer-specific loop links that are not observed in the dimer, this indicates a pronounced loss of activation loop flexibility in the dimer. The highly abundant loop link (K622–E624) within the β3-αC loop of the monomer disappeared completely in the dimer and was replaced by a high-confidence intermolecular cross-link between K622 in the β3-αC loop and E668 in the αD-helix. This observation is compatible with a face-to-face arrangement of the PKD kinase domain in the dimer, which has previously been observed experimentally for Chk2, the most closely related kinase to PKD for which a dimeric structure has been determined (22). Chk2 was therefore chosen as a template for comparative modeling of the PKD1 kinase domain dimer with experimental constraints derived from cross-linking mass spectrometry (XL–MS) using the Rosetta Comparative Modeling tool with C2 symmetry constraints (23). The resulting model revealed that the dimer interface additionally relies on a highly conserved acidic surface on the αG-helix consisting of E787, D788, and D791 (*SI Appendix*, Fig. S3*C*) that forms an intricate network of salt bridges and hydrogen bonds with R741 and S742 in the activation loop and N757 in the αE/F-αF loop (Fig. 1*G*). This is consistent with the stabilization of the activation loop indicated by XL–MS. To test this model, we introduced charge reversal mutations into the αG-helix (E787K) and the activation loop (R741E), which dramatically impaired dimerization of the kinase domain in solution (Fig. 1 *C* and *H* and *SI Appendix*, Fig. S3*D*). Activation loop phosphorylation on S742 or the mutagenesis of S738 and S742 to glutamate (PKD^KD^ 2E) also prevents dimerization (Fig. 1 *C* and *H*) (15), presumably due to the introduction of negative charge in the vicinity of the acidic αG-helix, which would lead to mutual repulsion. Single glutamate substitutions of either S738 or S742 are, however, not sufficient to dissociate the dimer (Fig. 1*C*).

In summary, the kinase domain of PKD forms a stable face-to-face dimer in solution mediated by a network of highly conserved electrostatic interactions. Intriguingly, however, the hydroxyl group of S742 is sequestered 15.8 Å away from the gamma phosphate of ATP assuming a *trans*-reaction and 13.1 Å away from the gamma phosphate of ATP assuming a *cis*-reaction, which makes this dimer incompatible with activation loop autophosphorylation and substrate binding. This observation prompted us to question what kinase domain dimerization is needed for in PKD1.

### Dimerization of the Kinase Domain Is Autoinhibitory.

**Fig. 2.**
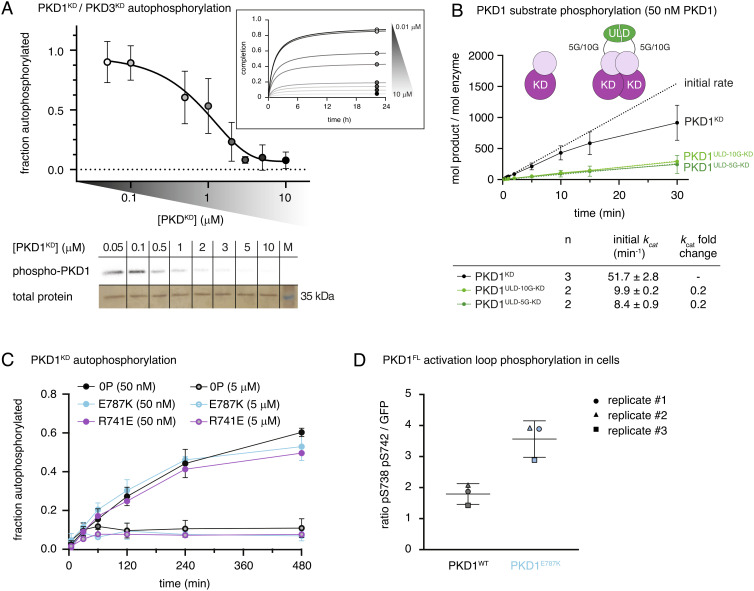
Dimerization of the kinase domain is autoinhibitory. (*A*) Radiometric autophosphorylation assay of PKD1^KD^ and PKD3^KD^. Error bars are the SD of two to four biologically independent experiments. *Inset*: Data points represent a reaction plateau at corresponding PKD^KD^ concentrations. *Bottom*: Representative autoradiograph of PKD1^KD^ samples compared to that of loading control (silver stain). (*B*) Substrate phosphorylation kinetics of PKD1^KD^ compared to those of ULD-mediated PKD1^KD^ dimers with either (Gly)_5_ or (Gly)_10_ linkers between the ULD and kinase domains. Assay performed at concentrations corresponding to monomeric PKD1^KD^. Error bars are the SD of *n* biologically independent experiments. Table indicates the number (n) of replicates and the values for *k_cat_* derived from a linear regression in the linear range. (*C*) Autophosphorylation kinetics of wild-type PKD1^KD^ compared to those of monomeric PKD1^KD^ E787K at low (50 nM) and high (5 μM) concentrations. Error bars are the SD of three biologically independent experiments. (*D*) PKD1 activation loop phosphorylation in HEK293T cells. Wild-type PKD1 (black) and PKD1 E787K (cyan).

To quantitatively assess the autocatalytic activity of the PKD kinase domain, we performed a series of autophosphorylation assays. In order to capture the influence of dimerization, different concentrations of PKD1^KD^ were chosen in the range of 50 nM to 10 µM so as to cover the dynamic range of the monomer–dimer equilibrium (Fig. 1*C*, black circles). Paradoxically for a reaction that was believed to occur in *trans*, autophosphorylation was more efficient with decreasing PKD concentration observed reproducibly for PKD1^KD^ and PKD3^KD^ (Fig. 2*A*). We ruled out the possibility that the decrease in activity was due to protein aggregation by dynamic light scattering analysis of PKD1^KD^ under the conditions of the assay (*SI Appendix*, Fig. S4 *A* and *B*). Furthermore, dilution of substoichiometrically phosphorylated PKD1^KD^ resulted in a subsequent increase in autophosphorylation (*SI Appendix*, Fig. S4*C*), indicating that the inactive protein is not aggregated. We therefore concluded that the kinase domains must dimerize in a manner that inhibits their ability to autophosphorylate. The affinity of homodimerization of the kinase domain on the basis of *trans*-autoinhibition was estimated to be 0.5 to 1.4 μM. To assess whether dimerization also affects substrate phosphorylation, we fused the homodimeric ULD of PKD1 (Fig. 1*C*) to its kinase domain with a polyglycine linker containing either five or ten glycine residues to enhance dimerization at a concentration (50 nM) at which the kinase domain alone is monomeric. Monitoring phosphate incorporation over time using a substrate peptide (Syntide-2), we observed a five-fold decrease in the initial catalytic rate in both ULD-PKD1^KD^ chimeras, consistent with the formation of an inhibitory dimer (Fig. 2*B*). Phosphorylation-induced dissociation of the kinase domains of PKD1 therefore has the potential to activate PKD simply by permitting substrate engagement.

To further investigate the impact of dimerization on PKD activity, we measured the autophosphorylation and substrate phosphorylation kinetics of the PKD1^KD^ E787K and R741E interface mutants. E787K was monomeric by SEC–MALS (Fig. 1*H*), while R741E exhibited a significant weakening of the dimer interface (*SI Appendix*, Fig. S3*D*). Both mutants showed comparable substrate phosphorylation kinetics to wild-type PKD1 (*SI Appendix*, Fig. S4*D*). Autophosphorylation at 50 nM was unaffected by the disruption of the interface (Fig. 2*C*), which demonstrates that the dimer we observe in solution does not drive autophosphorylation. Surprisingly, however, increasing concentrations of both interface mutants still resulted in inhibition of autophosphorylation that was indistinguishable from wild-type PKD1^KD^ (Fig. 2 *C* and *D*). These observations strengthen the notion that activation loop autophosphorylation does not occur in *trans*.

To further test whether molecular crowding could inhibit autophosphorylation by impeding productive collisions between PKD1^KD^ molecules, we made an inert kinase domain construct by combining a mutation of the catalytic aspartate (D706N) with S738E and S742E substitutions in the activation loop (PKD1^KD^ D706N/2E). This protein is dimerization deficient (Fig. 1*B*), catalytically inactive (*SI Appendix*, Fig. S4*E*), and cannot be phosphorylated. It is therefore an ideal mimic of PKD1^KD^ in both size and surface properties, while being unable to participate in the reaction. In contrast to both wild-type PKD1^KD^ and PKD1^KD^ E787K, increasing concentrations of PKD1^KD^ D706N/2E did not inhibit autophosphorylation of either PKD1^KD^ or PKD1^KD^ E878K (*SI Appendix*, Fig. S4*F*). These observations indicate that molecular crowding does not influence autophosphorylation but rather that an unphosphorylated activation loop is required for *trans*-autoinhibition.

Finally, we predicted that our dimer-disrupting mutant would lead to enhanced activation loop autophosphorylation in cells. To test this hypothesis directly, we transfected HeLa cells with expression constructs for full-length PKD1^WT^ and PKD1^E787K^. As expected, we observed an approximately twofold higher level of activation loop phosphorylation in unstimulated cells for the dimerization mutant, consistent with the inhibitory nature of PKD1 kinase domain dimerization (Fig. 2*D* and *SI Appendix*, Fig. S5*A*).

### PKD1 Activation Loop Phosphorylation Occurs in *Cis*.

**Fig. 3.**
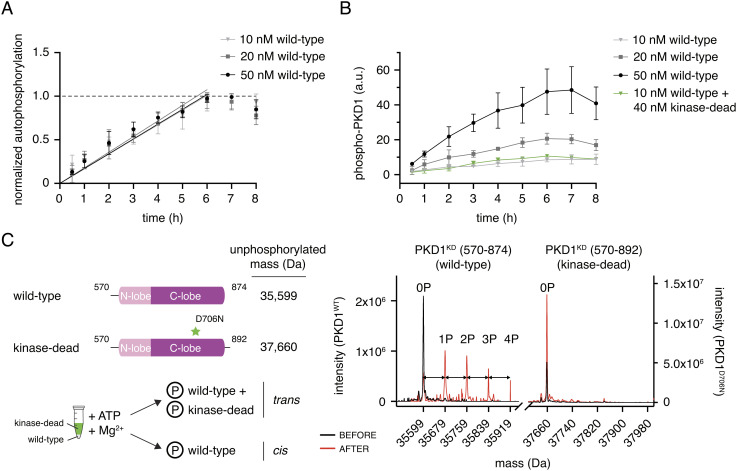
PKD1 activation loop autophosphorylation occurs in *cis*. (*A*) Activation loop autophosphorylation kinetics of PKD1^KD^ at low concentration. Error bars are the SD of three biologically independent experiments. (*B*) Activation loop autophosphorylation of kinase-dead PKD1^KD^ D706N in the presence and absence of excess wild-type PKD1^KD^. Error bars are the SD of three biologically independent experiments. (*C*) Mass spectrometry analysis of the autophosphorylation reaction containing 10 nM wild-type PKD1^KD^ and 40 nM kinase-dead PKD1^KD^ D706N shown in panel (*B*) Phosphospecies of wild-type PKD1^KD^ are separated by 80 Da (double-headed arrows).

Activation loop autophosphorylation in *cis* is highly controversial, and little data exist to support its possibility. Nevertheless, our observations prompted us to ask whether indeed it could be the mechanism by which PKD activates itself. A *cis*-reaction should be both concentration independent and limited by the intrinsic enzymatic rate of phosphotransfer, which should result in linear kinetics until the reaction reaches stoichiometric completion. To test whether this is the case for PKD1, we analyzed the kinetics of autophosphorylation over a range of PKD1^KD^ concentrations at which the kinase domain is not *trans-*autoinhibited (10 to 50 nM). The kinetics of phosphate incorporation were well approximated by a linear model at each concentration before reaching a plateau (Fig. 3*A*) and, when normalized by the amount of PKD1^KD^ in the reaction, exhibited identical rates (Fig. 3*A*). Since these findings strongly implied that the autophosphorylation reaction occurs in *cis*, we next asked whether or not wild-type PKD1^KD^ could phosphorylate a kinase-inactive PKD1^KD^ (D706N). Incubation of 10 nM wild-type PKD1^KD^ with an excess of 40 nM PKD1^KD^ D706N resulted in the incorporation of phosphate to a level that corresponds to 10 nM wild-type PKD1^KD^ and not to 50 nM total PKD1 (Fig. 3*B*). To confirm that the phosphate was incorporated exclusively into wild-type PKD1^KD^ and not kinase-inactive PKD1^KD^, we analyzed the reaction by mass spectrometry. To distinguish between the two constructs, we incubated a shorter wild-type PKD1^KD^ truncated in its C terminus by 18 amino acids (mass = 2,078 Da) with PKD1^KD^ D706N at a ratio of 1:4 and a total concentration of 50 nM. Mass spectrometry of the resulting mixture revealed the incorporation of up to four phosphates exclusively into wild-type PKD1^KD^ but not kinase-inactive PKD1^KD^ (Fig. 3*C*). These results unambiguously establish that activation loop autophosphorylation of PKD1 occurs in *cis* and not in *trans*.

The observation that the artificial dimerization of the PKD kinase domain by fusion to a dimerization domain accelerates autophosphorylation kinetics previously led us to propose PKD autophosphorylation in *trans*. To reconcile this observation with a *cis*-reaction, we repeated the mass spectrometry experiment with GST-tagged PKD1^KD^. Consistent with a *cis*-reaction, phosphorylation was restricted to the catalytically active PKD^KD^ (*SI Appendix*, Fig. S6).

### Activation Loop Phosphorylation Increases PKD Catalytic Activity.

**Fig. 4.**
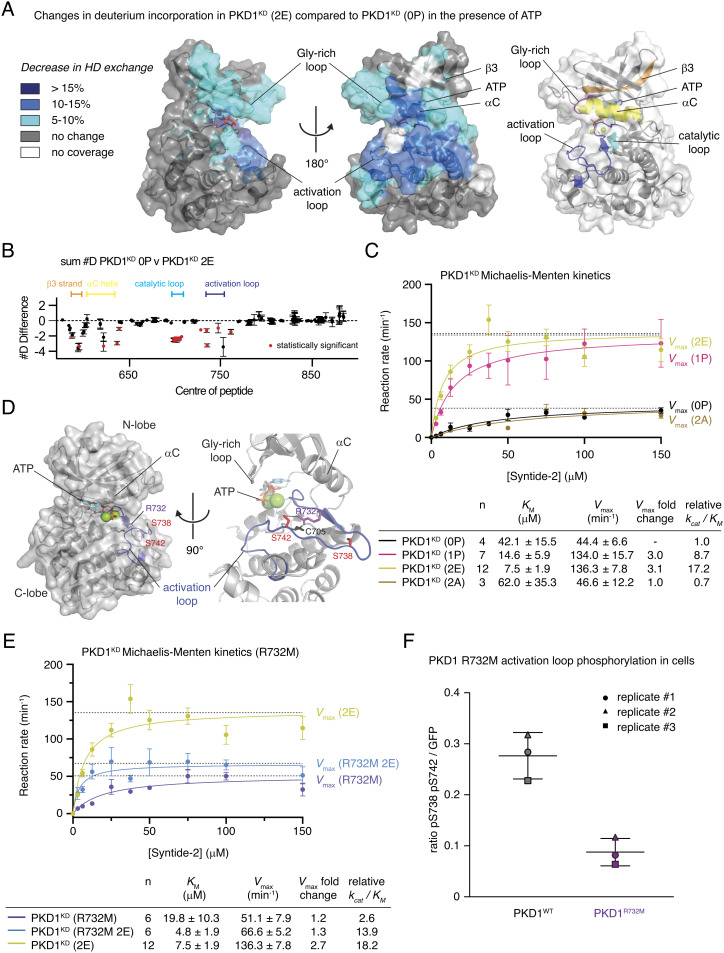
Activation loop autophosphorylation increases PKD1 catalytic activity. (*A*) Significant differences in deuterium incorporation in PKD1^KD^ (2E) compared to those in unphosphorylated PKD1^KD^ (0P) in the presence of ATP. Changes mapped onto the kinase domain of PKD1. Differences in exchange in a peptide were considered significant, if they met all three of the following criteria: ≥5% change in exchange and a ≥0.5-Da difference in exchange with a two-tailed *t* test value of less than 0.01 at any time point. *Right*: Color-coded cartoon of key structural elements that exhibit significant changes. Glycine-rich loop (magenta), strand β3 (orange), αC-helix (yellow), catalytic loop (cyan), and activation loop (blue). (*B*) The number of deuteron differences for all peptides analyzed over the entire deuterium exchange time course is shown, with each point representing an individual peptide (full exchange information for every peptide available in the source data). Statistically significant changes are indicated in red. (*C*) Michaelis–Menten kinetic analysis of PKD1^KD^ substrate phosphorylation. Unphosphorylated PKD1^KD^ (0P) (black), S742-phosphorylated PKD1^KD^ (1P) (magenta), PKD1^KD^ (2E) (yellow green), and PKD1^KD^ (2A) (brown). Error bars are the SD of *n* biologically independent experiments. Table indicates the number (*n*) of independent biological replicates and the values for *K_M_, V_max_*, and catalytic efficiency (*k_cat_*/*K_M_*) derived from fitting the data with the Michaelis–Menten equation. (*D*) Rosetta homology model of the PKD1 kinase domain indicating the arrangement of side chains surrounding S742 in the activation loop. Activation loop (blue) and phosphoacceptor residues (red). (*E*) Michaelis–Menten enzyme kinetics of PKD1^KD^ R732M (purple) compared to those of PKD1^KD^ (2E) (yellow green) and PKD1^KD^ R732M 2E (marine blue). Error bars are the SD of *n* biologically independent experiments. Table indicates the number (*n*) of independent biological replicates and the values for *K_M_, V_max_*, and catalytic efficiency (*k_cat_*/*K_M_*) derived from fitting the data with the Michaelis–Menten equation. (*F*) PKD1 activation loop phosphorylation in HEK293T cells. Wild-type PKD1 (black) and PKD1 R732M (purple).

Activation loop phosphorylation has long been identified as a crucial step in the activation of PKD (24), but the effect of this regulatory modification at the molecular level was only partially understood (25). We have shown in this (Fig. 1*B*) and previous work (15) that activation loop phosphorylation on S742 prevents kinase domain dimerization, which, in the context of an inhibitory kinase domain dimer, may be sufficient to activate PKD simply by relieving autoinhibition. Whether phosphorylation also increases the intrinsic catalytic activity of PKD is, however, unknown. To dissect the structural impact of activation loop phosphorylation, we used hydrogen–deuterium exchange mass spectrometry (HDX–MS) to compare the dynamics of PKD1^KD^ 0P to PKD1^KD^ 2E in the presence of both ATP and Mg^2+^. In PKD1^KD^ 2E compared to PKD1^KD^ 0P, we observed a strong and very specific decrease in the rates of HD exchange in the activation loop and the catalytic loop as well as the αC-helix and β1 and β3 strands (Fig. 4 *A* and *B*, and *SI Appendix*, Table S2 and Fig. S7*A*). Together, these elements constitute the kinase active site, and their stabilization is consistent with the stereotypical role of activation loop phosphorylation. Correspondingly, the catalytic efficiencies of stoichiometrically S742-phosphorylated PKD1^KD^ (1P) and PKD1^KD^ 2E were 9- and 17-fold higher, respectively, compared to those of unphosphorylated PKD1^KD^ (0P) (Fig. 4*C*).

Typically, kinases that depend on activation loop phosphorylation to acquire full activity rely on a conserved arginine in the so-called HRD motif to make electrostatic interactions with the respective phosphate group and thereby stabilize the activation loop in its active conformation. PKD, however, encodes a cysteine at this position (*SI Appendix*, Fig. S7*B*), which excludes it from the classical group of RD kinases. While it has been postulated that only RD kinases can be regulated by activation loop phosphorylation (8, 26), in silico modeling (Rosetta) of the PKD1^KD^ monomer revealed that R732 projects its guanidinium group into precisely the same three-dimensional position as the arginine in classical RD kinases (Fig. 4*D* and *SI Appendix*, Fig. S7*C*). Mutation of R732 to methionine in PKD1^KD^ abrogated the increase in *k_cat_* gained by phosphorylation or phosphomimetic substitutions (Fig. 4*E*), while neither the autophosphorylation nor substrate phosphorylation kinetics of unphosphorylated PKD1^KD^ R732M were significantly different to unphosphorylated, wild-type PKD1^KD^ (0P) (*SI Appendix*, Fig. S7*D* and Fig. 4*C*, respectively). Taken together, these data indicate that R732 in PKD1 plays an analogous role to the arginine of the classical HRD motif of eukaryotic protein kinases. When overexpressed in HEK293 cells, PKD1^R732M^ exhibited dramatically reduced activation loop phosphorylation (Fig. 4*F*, *SI Appendix*, Fig. S8), consistent with a defect in coordination of the phosphorylated activation loop and not a defect in autophosphorylation.

In summary, activation loop autophosphorylation activates PKD in two distinct ways: first by preventing autoinhibitory dimerization of its kinase domain and second by increasing its intrinsic catalytic activity.

### PKD Autophosphorylation and Substrate Phosphorylation Are Mechanistically Distinct.

**Fig. 5.**
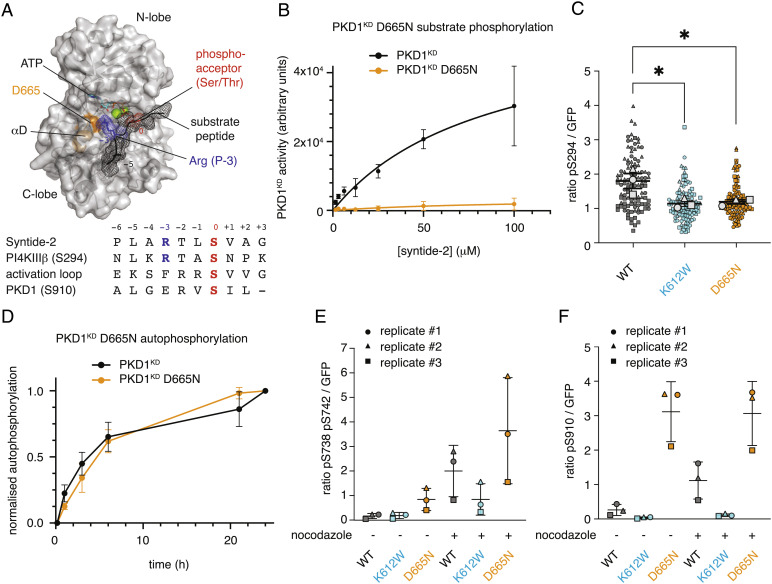
PKD1 autophosphorylation and substrate phosphorylation are mechanistically distinct. (*A*) Homology model of the PKD1^KD^–substrate interaction. Substrate peptide (black mesh), phosphoacceptor of substrate (red), Arg at the P-3 position (blue), and residue D665 in αD-helix (orange). *Below*: alignment of consensus sequence (Syntide-2), the PKD substrate PI4KIIIβ (S294), the PKD activation loop sequence (S742), and the C-terminal autophosphorylation motif (S910). (*B*) PKD1^KD^ (black) substrate phosphorylation kinetics compared to PKD1^KD^ D665N (orange). Error bars are the SD of two biologically independent experiments. (*C*) PKD1-mediated GPKDrep phosphorylation in HeLa cells determined by ratiometric imaging. Wild-type PKD1 (black), PKD1 K612W (blue), PKD1 D655N (orange). Data are represented in symbol-coded bee swarm SuperPlots, with each cell-level data point revealing which experiment it came from. Lines show mean and SEM of the replicate means. Statistical test: ordinary one-way ANOVA with Sidak’s multiple comparison test, *P < 0.05. (*D*) Autophosphorylation kinetics of wild-type PKD1^KD^ (black) compared to those of PKD1KD D665N (orange). Error bars are the SD of three biologically independent experiments. (*E*) PKD1 activation loop phosphorylation in HEK293T cells determined by western blot analysis. Wild-type PKD1 (black), PKD1 K612W (blue), and PKD1 D655N (orange). Lines show mean and SEM of the replicate means. (*F*) PKD1 S910 phosphorylation in HEK293T cells determined by western blot analysis. Wild-type PKD1 (black), PKD1 K612W (blue), and PKD1 D655N (orange). Lines show mean and SEM of the replicate means.

Substrates of PKD share a conserved consensus sequence (Syntide-2) that is not exhibited by either the phosphorylation motifs (S738 and S742) in the activation loop or an additional autophosphorylation motif in the C terminus of PKD1 and PKD2 (Fig. 5*A*). Kinases of the CAMK and AGC families specifically recognize an arginine in the P-3 position by virtue of a salt bridge to a conserved aspartate or glutamate residue (D665 in PKD1) in the β5-αD loop (Fig. 5*A*) (27). Mutation of D665 to asparagine (D665N) in PKD1 was previously reported to result in a loss of substrate specificity and consequent oncogenic rewiring but not a loss of activity (28). Purified, recombinant PKD1^KD^ D665N, however, was completely inactive against the PKD consensus substrate peptide Syntide-2 in vitro (Fig. 5*B*), and the activity of full-length PKD1^D665N^ against a Golgi-localized PKD activity reporter GPKDrep (29) was significantly reduced compared to that of wild-type PKD1 and similar to kinase-dead PKD1^K612W^ in cells (Fig. 5*C*). In vitro autophosphorylation of PKD1^KD^ D665N, on the other hand, was indistinguishable from wild-type PKD1^KD^ (Fig. 5*D*). We next investigated phosphorylation of PKD1^D655N^ in cells under basal conditions and after stimulation by nocodazole, which is known to strongly increase PKD activity (30). Interestingly, S738 and S742 in the activation loop and pS910 in the C terminus of PKD1 were hyperphosphorylated in PKD1^D665N^ in cells when compared to wild-type PKD1 and PKD1^K612W^ under basal conditions and with nocodazole stimulation. Notably, S910 phosphorylation, which was absent in PKD1^K612W^, increased further upon stimulation for wild-type PKD1 but not for PKD1^D665N^ (Fig. 5 *E* and *F* and *SI Appendix*, Fig. S9 *A* and *B*) (31) (15). Mutations which weaken the kinase domain dimer interface, including both E787K and D665N (*SI Appendix*, Fig. S3*D*), lead to activation loop hyperphosphorylation in cells (Fig. 2 *D* and 5 *E*). These observations indicate that activation loop autophosphorylation occurs in *cis*, and not *trans*, both in vitro and in cells. Mechanistically, activation loop autophosphorylation in *cis* and substrate phosphorylation in *trans* are distinct biochemical reactions.

### Substrate Binding-Deficient and Constitutively Dimeric PKD1 Block Secretion.

**Fig. 6.**
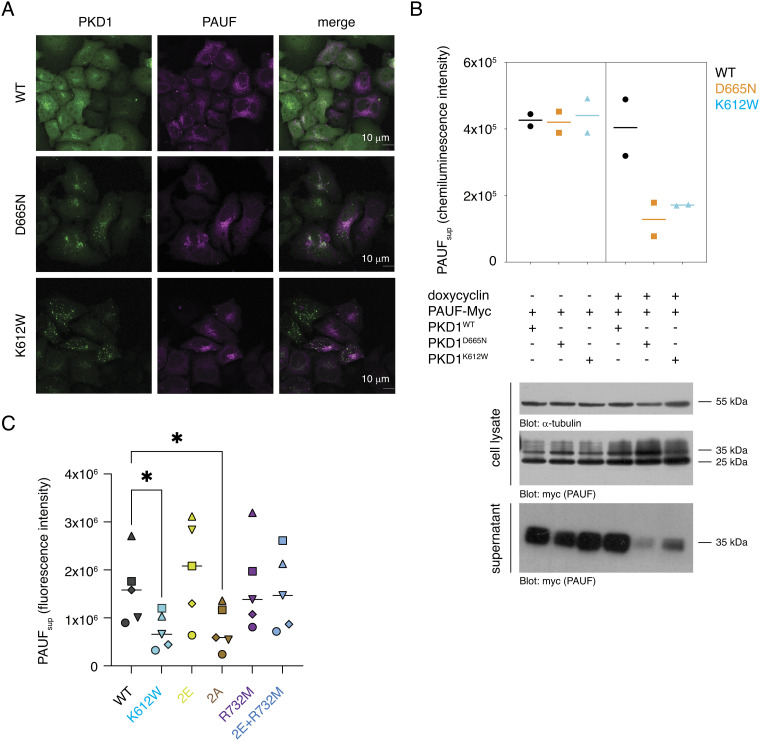
Substrate binding-deficient and constitutively dimeric PKD1 block secretion. (*A*) Subcellular localization of PKD1-GFP variants and PAUF in HeLa cells. (*B*) AUF secretion in FlpIN-TRex-HeLa cells under conditions of stable doxycycline-inducible PKD1 expression and transient PAUF expression. Lines show mean of replicate data. (*C*) PAUF secretion in HEK293T cells under conditions of transient PKD1 and PAUF expression. Lines show mean of replicate data. Statistical test: RM one-way ANOVA with Sidak’s multiple comparison test, *P < 0.05.

PKD controls the fission of basolateral membrane-targeted cargo vesicles from the TGN (10). To investigate the impact of the substrate phosphorylation-deficient D665N mutant on protein secretion, we monitored the distribution of pancreatic adenocarcinoma up-regulated factor (PAUF), a known PKD-dependent cargo. PAUF secretion is arrested in the TGN by overexpression of kinase-dead PKD^K612W^ (10). Consistent with the loss of substrate phosphorylation, but not autophosphorylation, we observed the same Golgi accumulation of PAUF in cells transfected with PKD1^D665N^ (Fig. 6*A* and *SI Appendix*, Fig. S10*A*), while PAUF secretion was completely abrogated (Fig. 6*B*). This further strengthens the essentiality of PKD in secretion, wherein it controls the fission of cargo vesicles from the TGN.

We previously demonstrated that activation loop phosphorylation prevents kinase domain dimerization, thereby relieving autoinhibition in addition to increasing catalytic activity (Fig. 1 *C* and 4 *C*). Conversely, a mutant of the kinase domain in which both phosphorylation sites are mutated to alanine (PKD1^KD^ 2A) is dimeric in vitro (Fig. 1*C*). This suggests that PKD that is unable to be phosphorylated might exert a dominant negative effect on secretion in cells due to constitutive autoinhibition. Indeed, PKD1^2A^ abrogated PAUF secretion (Fig. 6*C* and *SI Appendix*, Fig. S11 *A*–*C*), despite the fact that unphosphorylated PKD1^KD^ (0P) has substantial basal activity in vitro (Fig. 4*C*) and the protein is ectopically overexpressed. In contrast, expression of PKD1^R732M^, which has the same kinetics of substrate phosphorylation as PKD1^2A^ in vitro (Fig. 4 C and E), does not impair PAUF secretion (Fig. 6*C* and *SI Appendix*, Fig. S11 *A*–*C*). This is presumably due to dissociation of the kinase domain dimer upon low, but not absent, activation loop phosphorylation in stimulated cells (Fig. 4*F*). Consistent with this, while activation loop phosphomimetics combined with R732M results in reduced catalytic activity in vitro (Fig. 4*E*), they still prevent inhibitory dimerization of the kinase domain (Fig. 1*C*). Together, these observations strengthen the physiological relevance of a face-to-face autoinhibitory dimer of PKD.

### Autoregulation of Membrane Binding Depends on ATP but Not Dimerization.

Previous work has established that the DAG-binding C1 domains of PKD are sequestered in its autoinhibited, cytosolic conformation (15). The endogenous concentration of PKD has been estimated to be 3 to 32 nM (15, 31). Dimerization of PKD via both its ULD and kinase domains, which have affinities of homodimerization of 0.5 to 2 µM and 0.9 µM, respectively, suggests that PKD is constitutively dimeric in cells and autoinhibited in the absence of DAG. Indeed, calibrated size exclusion chromatography of endogenous PKD is consistent with the expected molecular weight of a dimer (32). To test whether dimerization is relevant for the regulation of DAG binding, we examined membrane translocation kinetics as a proxy for the stability of the autoinhibited state. We employed a ratiometric membrane translocation assay in which we measure the rates of translocation of wild-type and mutant PKD variants to the plasma membrane upon treatment of the cells with phorbol ester, a natural DAG mimetic. Since the binding to phorbol esters is essentially irreversible, the rate of translocation is correlated with the accessibility of the phorbol ester-binding C1 domains. By cotransfecting spectrally separable fluorescent fusion proteins, wild-type and mutant PKD variants can be compared within the same cell (33, 34) (*SI Appendix*, Fig. S12*A*). Disruption of kinase domain dimerization either by introduction of phosphomimetics in the activation loop (2E) or by mutation of the αG interface (E787K) resulted in membrane translocation kinetics that were indistinguishable from PKD1^WT^ (*SI Appendix*, Fig. S12 *B* and *C*). Finally, we combined a mutation in the ULD dimer interface (F104E in PKD1 and F59E in CeDKF1) (Fig. 1*C*) (15) with the kinase domain 2E mutant (PKD1^F104E+S738E/S742E^) to create a construct which is compromised in all known dimerization interfaces. This construct, which is presumably monomeric, also did not show altered membrane translocation kinetics (*SI Appendix*, Fig. S12*D*). We therefore conclude that the autoregulation of DAG binding does not intrinsically depend on PKD dimerization.

Previous studies have shown an enrichment of kinase-dead PKD at the TGN when compared to that of wild-type PKD (10). Intriguingly, while mutation of the ATP-binding lysine, K612, resulted in accelerated translocation kinetics (*SI Appendix*, Fig. S12*E*), mutation of the catalytic aspartate in the active site to asparagine (D706N) had no effect on membrane binding (*SI Appendix*, Fig. S12*F*). These observations indicate that ATP binding, but not catalysis, is important for stabilization of the intramolecular, autoinhibited assembly. Interestingly, PKD1^D665N^, which exhibited a similar enrichment at the TGN, also translocated to the plasma membrane significantly faster than wild-type PKD1 (*SI Appendix*, Fig. S12*G*). In addition to mediating substrate binding, D665 is predicted to make hydrogen bonds to the ribose moiety of ATP, suggesting that nucleotide binding is critical for stable PKD autoinhibition.

## Discussion

**Fig. 7.**
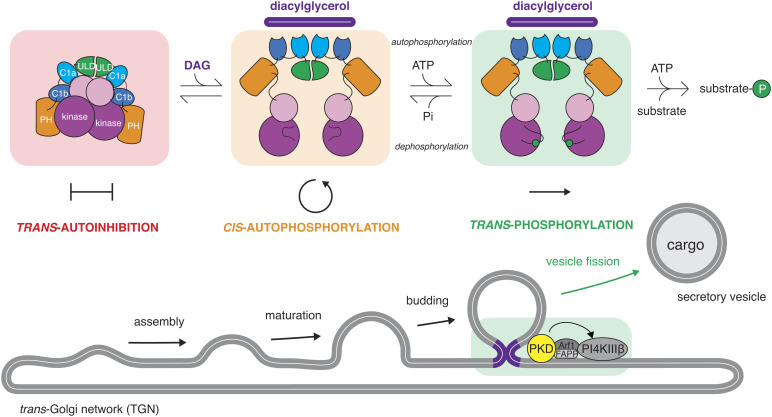
PKD autoinhibition in *trans* regulates activation loop autophosphorylation in *cis.* Model for the control of PKD by DAG binding and subsequent triggering of cargo vesicle fission from the TGN. PKD adopts a constitutively dimeric conformation in the cytosol of cells in which its kinase domains are maintained in a *trans*-autoinhibitory face-to-face dimer. Binding to DAG at the TGN, presumably in the vicinity of the bud neck of cargo vesicles, results in conformational changes that relieve the inhibitory dimerization of its kinase domains, leading to activation loop *cis*-autophosphorylation. Activation loop phosphorylation both promotes *trans*-phosphorylation of substrates and prevents reassembly of the kinase domains.

The majority of eukaryotic protein kinases that autoactivate by activation loop phosphorylation are believed to do so in *trans*. This necessitates their transient dimerization. In this study, we demonstrate that kinases can be regulated by the inverse mechanism: dissociation of an inhibitory dimer followed by autophosphorylation in *cis* (Fig. 7). While our study focuses specifically on the regulation of PKD, *cis*-autophosphorylation of the activation loop is likely to be a much more common mode of kinase regulation.

Our findings lead to a revised model of PKD regulation (Fig. 7). In the absence of activating signals, PKD is maintained in a dimeric, autoinhibited conformation mediated by homodimerization of its ULD and kinase domains. The inactive conformation is characterized by a face-to-face arrangement of its kinase domains that inhibits both activation loop autophosphorylation and substrate phosphorylation. The inactive conformation of PKD sequesters its regulatory C1 domains in intramolecular interactions that inhibit binding to DAG in the membranes of the TGN. Upon DAG enrichment in the TGN, the C1 domains disengage from the intramolecular assembly, triggering conformational changes that are favorable for activation loop autophosphorylation in *cis*. Once phosphorylated, the kinase domain of PKD acquires full catalytic activity and is unable to dimerize, thereby protecting it from *trans*-autoinhibition. PKD is thereby fully primed to initiate downstream signaling, resulting in cargo secretion from the TGN.

PKD activation loop phosphorylation has long been thought to occur in *trans* on the basis that catalytically inactive PKD expressed in cells acquires S738/S742 activation loop phosphorylation, presumably via endogenous PKD (14). Our own recent discovery of the ULD dimerization domain in the *C. elegans* PKD homologue, Dkf1, combined with accelerated autophosphorylation kinetics of a dimeric GST–PKD1^KD^ fusion protein led us to conclude that this mediates phosphorylation in *trans* (15). This conclusion, while logical and commonly accepted as evidence for *trans*-autophosphorylation, was incorrect as GST-mediated dimerization in fact accelerates *cis*-autophosphorylation.

Activation loop autophosphorylation in *cis* was long considered to be impossible on the basis that the activation loop runs in the opposite direction to a canonical substrate peptide. While some kinases have been reported to autophosphorylate in *cis* via a variety of mechanisms (35), rationalization of such mechanisms at the atomic level is still missing. However, many receptor tyrosine kinases, including the insulin receptor kinase (IRK) and fetal liver tyrosine kinase (FLT3), can adopt a conformation in which the activation loop tyrosine, which undergoes autophosphorylation, superimposes perfectly with the phosphoacceptor tyrosine of a substrate peptide (36–38). While these structures have been interpreted to represent an autoinhibited conformation, they provide compelling evidence that a *cis*-arrangement of the activation loop is physically possible.

We have shown that the mechanisms of autophosphorylation and substrate phosphorylation in PKD are mechanistically distinct. However, the kinetics of PKD *cis*-autophosphorylation are extremely slow and not restricted to monophosphorylation of Ser742, suggesting that PKD relies on additional activating inputs for precise and efficient autoactivation. The acceleration elicited by GST-mediated dimerization demonstrates the activation potential of the *cis*-reaction. Such a stimulus may come from the regulatory domains of PKD itself or other interaction partners. Equally, the relief of dimerization-mediated autoinhibition of PKD presumably relies on conformational changes in its regulatory domains upon membrane binding that will require further investigation.

Since activation loop autophosphorylation prevents autoinhibition in *trans*, the reaction must be prohibited in the absence of an activating stimulus. In the context of a *cis*-reaction, the slow kinetics perhaps safeguard the kinase domain from spurious activation during transient dissociation events. Once phosphorylated, however, activation loop dephosphorylation is a prerequisite for reestablishing kinase domain-mediated PKD autoinhibition, although the phosphatase that inactivates PKD remains to be identified.

Autoinhibition is common to many protein kinases and serves to inactivate them in the absence of a stimulus. The αG-helix is at the center of a surface epitope commonly employed in the regulation of kinase activity (39–45). αG-mediated, autoinhibitory face-to-face dimers of the related death-associated protein kinases (DAPKs) have previously been observed (46, 47), yet these kinases do not autophosphorylate (48). Whereas the DAPKs are obligate homodimers by virtue of their C-terminal coiled coil and PKD is likely an obligate ULD-mediated homodimer, the closest related kinase to PKD, checkpoint-associated kinase 2 (Chk2), undergoes regulated dimerization. Phosphorylation of Thr68 by the ataxia telangiectasia-mutated (ATM) kinase in response to DNA damage promotes Chk2 dimerization via its forkhead-associated (FHA) domain (49–52). The structure of a construct of Chk2 containing both its FHA and kinase domains revealed that FHA-mediated dimerization of Chk2 drives face-to-face apposition of its kinase domains in an arrangement that was interpreted to represent the *trans*-autophosphorylation reaction (22). A similar structure has been obtained for the yeast homologue of Chk2, Rad53 (53). GST-mediated dimerization of the Chk2 kinase domain has also been shown to enhance activation loop phosphorylation (54). Intriguingly, however, the arrangement of the kinase domains in both structures is remarkably similar to that observed in autoinhibited DAPK2 and the PKD dimer presented in this study. Further work will undoubtedly be required to reconcile the differences between dimerization-driven DAPK and PKD autoinhibition and dimerization-mediated activation of Chk2.

Activation loop phosphorylation leads to increased catalytic activity of PKD, despite its classification as a non-RD kinase. However, phosphorylation also serves another, essential, purpose, which is to prohibit inhibitory dimerization. Expression of PKD that cannot be phosphorylated in its activation loop, but which retains significant basal activity, results in a similar dominant negative effect to the expression of kinase-dead PKD. The embryonic lethal phenotype of homozygous S744A/S748A PKD1 knock-in mice (55) may, therefore, reflect the inability of DAG to relieve *trans*-autoinhibition of PKD1 via activation loop autophosphorylation upon binding to the TGN in addition to the reduction in intrinsic catalytic activity. Such an interpretation, however, will require further experimental corroboration.

## Materials and Methods

### Cross-Linking Coupled to Mass Spectrometry (XL–MS).

#### In-solution cross-linking.

Cross-linking of PKD1^KD^ (0P) was performed in 40 mM MES pH 6.5 and 100 mM NaCl. 4 µM protein containing 1 mM ATP and 2 mM MgCl_2_ was mixed with varying concentrations of EDC and Sulfo-NHS at a constant ratio of 1:2.5 and incubated for 30 min in the dark. The reaction was quenched with 20 mM β-mercaptoethanol and 50 mM Tris pH 7.5. Cross-linked monomeric and dimeric species were separated by SDS–PAGE. Details of the XL–MS sample analysis can be found in *SI Appendix*.

### In Silico Modeling.

Homology modeling of the PKD1 kinase domain (residues 576 to 873) was performed using the Rosetta Comparative Modeling tool (20) and the structures of phosphorylase kinase (PDB 2phk), Chk2 (PDB 3i6u), and DAPK2 (PDB 2a2a; 2yaa) as templates. The active state was modeled using ligand coordinate and parameter files for ATP calculated for ATP from the active conformation of phosphorylase kinase (PDB 2phk) and the built-in Rosetta parameter file for magnesium. The model was compared to the AlphaFold2 (21) prediction for the PKD1 kinase domain and tested biochemically.

Homology modeling of the PKD1 kinase domain dimer was performed using the Rosetta Comparative Modeling tool and symmetry imposed by the structure of Chk2 (PDB 3i6u). Additional constraints were imposed based on dimer-specific intramolecular EDC cross-links (615 to 622) and intermolecular EDC cross-links (622 to 831) and an EDC cross-link common to both monomer and dimer (616 to 624), with a distance constraint of 3.8 Å. The model was tested biochemically by introducing a single point mutation (E787K) into the predicted dimer interface.

### Size Exclusion Chromatography Coupled to Multiangle Light Scattering (SEC–MALS).

For determination of particle mass and polydispersity, 60 µL of purified PKD1^KD^ at 2 mg/mL was injected onto an S200 10/300 column (Cytiva) connected to a 1260 Infinity HPLC (Agilent Technologies). A miniDAWN TREOS (Wyatt) was used to detect light scattering at 690 nm, and a Shodex RI-101 (Shodex) detector was used for refractive index measurement. All runs were performed at room temperature in 20 mM HEPES pH 7.4, 150 mM KCl, 1 mM EDTA, 1 mM TCEP, and 1% (v/v) glycerol.

### Kinase Assays.

All kinase assays were done with radiolabeled [γ-32P] ATP (Hartmann Analytic) in a reaction buffer containing 50 mM HEPES pH 7.4, 150 mM KCl, 1 mM TCEP, 1 mM EDTA, 1% (v/v) glycerol, 0.05% (v/v) Tween-20, 1 mM ATP, 5 mM MgCl_2_, and 1 µL [γ-32P] ATP/100 µL reaction and were terminated by the addition of 10 mM EDTA. The radioactivity from phosphorylated material immobilized in polyacrylamide gels or on 0.45-μm nitrocellulose membranes (Cytiva) was detected by exposure to a phosphor screen, imaged by an Amersham Typhoon phosphorimager, quantified in ImageJ, and converted into molar quantities by calibration with internal standards. Details of the different kinase assays performed can be found in *SI Appendix*.

### HDX–MS.

#### Sample preparation.

HDX reactions for wild-type PKD1^KD^ and PKD1^KD^ S738/742E were conducted in a final reaction volume of 6 µL with a final concentration of 5 µM (30 pmol) PKD1^KD^ or PKD1^KD^ S738/742E. The reaction was initiated by the addition of 5.25 µL of D_2_O buffer (20 mM pH 7.5 HEPES, 100 mM NaCl, 1 mM ATPγS, 5 mM MgCl_2_, and 85.8% D_2_O (V/V)) to 0.75 µL of PKD1cat WT or mutant (final D_2_O concentration of 71.3%). The reaction proceeded for 3, 30, 300, or 3,000 s at 20 °C before being quenched with ice-cold acidic quench buffer, resulting in a final concentration of 0.6 M guanidine HCl and 0.9% formic acid postquench. All conditions and time points were generated in triplicate. Samples were flash frozen immediately after quenching and stored at −80 °C until injected onto the ultraperformance liquid chromatography (UPLC) system for proteolytic cleavage, peptide separation, and injection onto a QTOF for mass analysis, described below. Details of the HDX–MS analysis can be found in *SI Appendix*.

### Cell Culture and Transfection.

HeLa and HEK293T cells were maintained in RPMI 1640 medium supplemented with 10% FCS. Cell lines were authenticated using Multiplex Cell Authentication by Multiplexion (Heidelberg, Germany) as described recently (Castro et al., 2013). The SNP profiles matched known profiles or were unique. Cells were tested negative for *mycoplasma* contamination using MycoAlert (Lonza, Switzerland). For transient plasmid transfections, HEK293T cells were transfected with TransIT-293 (Mirus Bio). HeLa cells were transfected with TransIT-HeLaMONSTER (Mirus Bio).

Flp-In T-REx-HeLa cells (generated by Elena Dobrikova and Matthias Gromeier, Duke University Medical Center, Durham, NC, USA) were grown in DMEM containing 10% FCS, 100 µg/mL zeocin, and 10 µg/mL blasticidin (HeLa). These cells stably express the Tet repressor, contain a single Flp Recombination Target (FRT) site, and were used to generate the Flp-In-T-REx-PKD1-GFP lines. Cells were cotransfected with pcDNA5/FRT/TO-PKD1-GFP and the Flp recombinase expression plasmid pOG44 at a ratio of 1:10 and then selected with 500 µg/mL hygromycin B. Induction of protein expression with doxycycline was at 10 ng/mL. Details of the immunofluorescence staining and confocal microscopy can be found in *SI Appendix*.

### PAUF Secretion Assay.

FlpIN Trex HeLa cells stably expressing PKD1-EGFP variants WT, K612W, and D655N were transfected with a plasmid encoding PAUF-Myc-His, and after another 24 h, the expression of PKD1-EGFP was induced with doxycycline. HEK293T cells were transiently transfected with plasmids encoding PKD1-EGFP variants WT, S2E, S2A, R732M, or R732M S2E and PAUF-Myc-His in a ratio of 1:5. Growth medium was removed 24 h posttransfection, and cells were incubated in serum-free medium. After 5 h, the medium was collected, and cells were lysed. Aliquots of medium and detergent-soluble cell proteins were assayed for PAUF content by western blot analysis.

## Supplementary Material

Appendix 01 (PDF)

## Data Availability

[Proteomics] data have been deposited in [PRIDE] (PXD031997, PXD033139). All study data are included in the article and/or *SI Appendix*.
