# Supplementary material for: PKD autoinhibition in trans regulates activation loop autophosphorylation in cis
Source: Proc Natl Acad Sci U S A. 2023 Feb 6;120(7):e2212909120. doi: 10.1073/pnas.2212909120 (PMC9962925; doi:10.1073/pnas.2212909120)
Supplement: Supplementary file 1 — Appendix 01 (PDF) [file pnas.2212909120.sapp.pdf]

PKD autoinhibition in *trans* regulates activation loop autophosphorylation in *cis*

Ronja Reinhardt[1,2], Kai Hirzel[3], Gisela Link[3], Stephan A. Eisler[4], Tanja Hägele[3], Matthew A.H. Parson[5], John E. Burke[5,6], Angelika Hausser[3,4] and Thomas A. Leonard[1,2,7]

[1]Department of Structural and Computational Biology, Max Perutz Labs, Campus Vienna Biocenter 5, 1030 Vienna, Austria

[2]Department of Medical Biochemistry, Medical University of Vienna, 1090 Vienna, Austria

[3]Institute of Cell Biology and Immunology, University of Stuttgart, Allmandring 31, 70569 Stuttgart, Germany

[4]Stuttgart Research Center Systems Biology, University of Stuttgart, Nobelstrasse 15, 70569 Stuttgart, Germany

[5]Department of Biochemistry and Microbiology, University of Victoria, Victoria, BC, Canada V8W 2Y2

[6]Department of Biochemistry and Molecular Biology, The University of British Columbia, Vancouver, British Columbia V6T 1Z3, Canada

[7]Correspondence: thomas.leonard@meduniwien.ac.at

Supplementary Information

Supplementary Materials and Methods
Supplementary References
Supplementary Figures S1-S12
Supplementary Tables S1-S2

Supplementary Materials and Methods

Reagents, antibodies and plasmids

The plasmid encoding G-PKDrep (1) and the plasmids encoding GFP-tagged wild-type PKD1, PKD1 K612W, PKD1 S738/S742E and PKD1 S738/S742E (2) were described previously. R732M, E787K and D665N mutations were generated by site-directed mutagenesis and plasmids were verified by sequencing. The plasmid encoding PAUF-myc/his was kindly provided by Vivek Malhotra (CRG, Barcelona, Spain). The pSer294-specific rabbit polyclonal antibody used for detection of G-PKDrep phosphorylation has been previously described (1). Commercially available antibodies used were as follows: anti-phospho-PKD (Ser744/748) rabbit polyclonal antibody, anti-phospho PKD (Ser 916) rabbit polyclonal antibody, anti-myc mouse monoclonal antibody clone 9B11 (all from Cell Signaling Technologies), anti-GFP mouse monoclonal antibody (Roche Diagnostics), anti-tubulin α mouse monoclonal antibody (Merck Chemicals GmbH), and anti-p230 mouse monoclonal antibody (BD Biosciences). Secondary antibodies used were Alexa546 or Alexa488 coupled goat anti–mouse immunoglobulin G (IgG) (Life Technologies), and horseradish peroxidase (HRP) coupled goat anti–mouse and anti–rabbit IgG (Dianova). Nocodazole and doxycyline were purchased from Sigma-Aldrich.

Protein expression and purification

TEV-cleavable N-terminal GST fusion constructs of PKD1^{KD} (570-892), PKD3^{KD} (563-890), PKD1$^{ULD\text{-}5G\text{-}KD}$ (48-143-(Gly)$_5$-570-892), PKD1$^{ULD\text{-}10G\text{-}KD}$ (48-143-(Gly)$_{10}$-570-892), and short PKD1^{KD} (570-874) were cloned into the pFastBac Dual vector for expression in Sf9 insect cells. Mutant constructs of PKD1^{KD} were generated by site directed mutagenesis.

Sf9 cell pellets from 1L culture were lysed in 100 mL lysis buffer (50 mM Tris pH 7.5, 150 mM KCl, 1 mM TCEP, 1 mM EDTA, 1 mM EGTA, 0.25 % CHAPS, 20 mM benzamidine, 1 mM PMSF, 4mM $MgCl_2$, 1U benzonase, 1x protease inhibitor cocktail (Sigma P8849)). After centrifugation (38 397 g, 4°C, 30 min) the cleared cell lysate was incubated with approximately 3 mL glutathione sepharose beads (Cytiva) for 2 h at 4°C. The beads were then washed with buffer A (50 mM Tris pH 7.5, 150 mM KCl, 1 mM TCEP, 1 mM EDTA, 1 mM EGTA, 0.25 % CHAPS) and dephosphorylated over night at 4°C with 2.2 nmol lambda-phosphatase (purified in house) in 4 mM $MnCl_2$. The beads were washed multiple times in buffer A with varying salt concentrations (2x 400mM KCl, 2x 40mM, 2x

150mM), before cleavage with 8.8 nmol TEV protease (purified in house) for 2h at room temperature. Cleaved PKD constructs in solution were separated from the beads, diluted to 37.5 mM KCl in buffer Q_A (50 mM Tris pH 8.5, 1 mM TCEP, 1 mM EDTA, 1 % glycerol) and bound to a HiTrap Q HP anion exchange column (Cytiva). After gradient elution with buffer Q_B (50 mM Tris pH 8.5, 1 M KCl 1 mM TCEP, 1 mM EDTA, 1 % glycerol) the peak fractions were pooled, concentrated, and injected on a S200 increase 10/300 GL size exclusion column (Cytiva) that was equilibrated in 20 mM Tris pH 7.5, 150 mM KCl, 1 mM TCEP, 1 mM EDTA, 1 % (v/v) glycerol. Constructs with mutated activation loop phosphorylation sites were not treated with lambda phosphatase.

Stoichiometric phosphorylation of $PKD1^{KD}$ (1P) was achieved by incubating the washed, GST-$PKD1^{KD}$ bound beads with 1mM ATP and 5 mM $MgCl_2$ at 4°C overnight followed by 1 h at room temperature. After TEV cleavage, the phosphorylated species were separated on a MonoQ 5/50 GL column (Cytiva) and characterized by intact mass spectrometry.

$PKD1^{ULD}$ proteins were purified according to (3).

Analytical size exclusion chromatography

A Superdex 200 increase 3.2/300 size exclusion column (Cytiva), connected to an Äkta Pure with a Micro configuration (Cytiva) was equilibrated in buffer (20 mM HEPES pH 7.4, 150 mM KCl, 1 mM EDTA, 1 mM TCEP, 1 % (v/v) glycerol) at 4°C. 25 µL protein samples were clarified by centrifugation (5 min, 21 000 g) prior to injection at constant flow rate. Peak elution volumes are reported, while peak concentration was determined by integrating the peak area for 20 µL centered on the highest point.

Differential scanning fluorimetry (DSF)

Thermal stability measurements of $PKD1^{KD}$ were performed in 20 mM Tris pH 7.5, 150 mM KCl, 1 mM TCEP, 1 mM EDTA, 1% (v/v) glycerol with 5x SYPRO Orange (life technologies) using a BioRad iQ™5 Multicolor Real-Time PCR Detection System. A nucleotide-free control was compared to samples containing 5 mM $MgCl_2$ and 1 mM ATP or ADP. Each condition was measured at four protein concentrations (1.25, 2.5, 5, 10 µM) and, in the absence of a concentration-dependent change in melting temperature, the measurements were averaged and presented as replicates.

Mass spectrometry and data analysis

Sample preparation for mass spectrometry

The Coomassie-stained gel band was de-stained with a mixture of acetonitrile (Chromasolv®, Sigma-Aldrich) and 50 mM ammonium bicarbonate (Sigma-Aldrich). The proteins were reduced using 10 mM dithiothreitol (Roche) and alkylated with 50 mM iodoacetamide. Trypsin (Promega; Trypsin Gold, Mass Spectrometry Grade) was used for proteolytic cleavage. Digestion was carried out with trypsin at 37°C overnight. Formic acid was used to stop the digestion and the extracted peptides were desalted using C18 Stagetips (4).

Sample analysis

Peptides were analyzed on an UltiMate 3000 HPLC RSLC nanosystem (Thermo Fisher Scientific) coupled to a Q Exactive HF-X, equipped with a nano-spray ion source using coated emitter tips (PepSep, MSWil). Samples were loaded on a trap column (Thermo Fisher Scientific, PepMap C18, 5 mm × 300 µm ID, 5 µm particles, 100 Å pore size) at a flow rate of 25 µL min^{-1} using 0.1% TFA as mobile phase. After 10 min, the trap column was switched in-line with the analytical C18 column (Thermo Fisher Scientific, PepMap C18, 500 mm × 75 µm ID, 2 µm, 100 Å) and peptides were eluted applying a segmented linear gradient from 2% to 80% solvent B (80% acetonitrile, 0.1% formic acid; solvent A 0.1% formic acid) at a flow rate of 230 nL/min over 120 min. The mass spectrometer was operated in data-dependent mode, survey scans were obtained in a mass range of 350-1600 m/z with lock mass activated, at a resolution of 120,000 at 200 m/z and an AGC target value of 1E6. The 15 most intense ions were selected with an isolation width of 1.2 Thomson for a max. of 150 ms, fragmented in the HCD cell at stepped normalized collision energy at 26%, 28%, and 30%. The spectra were recorded at an AGC target value of 1E5 and a resolution of 60,000. Peptides with a charge of +1, +2, or >+7 were excluded from fragmentation, the peptide match feature was set to preferred, the exclude isotope feature was enabled, and selected precursors were dynamically excluded from repeated sampling for 20 seconds within a mass tolerance of 8 ppm.

Raw data were processed using the MaxQuant software package 1.6.17.0 (5) and searched against the Uniprot human reference proteome (January 2020, www.uniprot.org) as well as a database of most common contaminants. The search was performed with standard identification settings: full trypsin specificity allowing a

maximum of two missed cleavages. Carbamidomethylation of cysteine residues was set as fixed, oxidation of methionine and acetylation of protein N-termini as variable modifications. All other settings were left at default. Results were filtered at a false discovery rate of 1% at protein and peptide spectrum match level. To identify cross-linked peptides, the spectra were searched using pLink software 2.3.9 (6) against the sequences of the top 8 non-contaminant proteins from the MQ search sorted by iBAQ. Carbamidomethylation of cysteine was set as fixed, oxidation of methionine and acetylation of protein N-termini as variable modifications. The enzyme specificity was set to trypsin allowing 4 missed cleavage sites. Crosslinker settings were selected as EDC. Search results were filtered for 1% FDR (false discovery rate) on the PSM level (peptide-spectrum matches) and a maximum precursor mass deviation of 5 ppm. To remove low quality PSMs, additionally an e-Value cutoff of < 0.001 was applied. Cross-link maps were generated in xiNET (7).

Proteomics data deposition

The mass spectrometry proteomics data have been deposited to the ProteomeXchange Consortium via the PRIDE partner repository (8) with the dataset identifier PXD031997.

Kinase assays

Assays probing the fraction of autophosphorylated PKD^{KD} at the reaction plateau at different concentrations were performed at 30°C overnight. After termination of the reaction, aliquots corresponding to 0.5 pmol of PKD^{KD} were subjected to SDS-PAGE to separate the phosphorylated protein from excess [γ-32P] ATP. The gels were stained with silver stain (Invitrogen), dried and exposed to a phosphor screen overnight. The fraction of phosphorylated PKD^{KD} was calculated from the internal standard, assuming a single phosphate is incorporated per PKD^{KD} molecule (confirmed by intact mass spectrometry). To test the reversible nature of the plateau, a reaction containing PKD^{KD} was left to reach completion over 2 h at 30°C. A fraction was then diluted to a final concentration of 50 nM and allowed to react for an additional 18 h under the same conditions. Aliquots corresponding to 0.5 pmol of each reaction were subjected to SDS-PAGE and analyzed as described above.

For autophosphorylation assays probing the *trans*-autoinhibitory potential of PKD1^{KD} (D706N/2E), 50 nM of active PKD1^{KD} (0P) or PKD1^{KD} (E787K) was mixed with 0.5 µM or 5 µM of kinase dead PKD1^{KD} (D706N/2E), and incubated over night at 30°C. Aliquots corresponding to 0.5 pmol of active kinase were subjected to SDS-PAGE. The gels were washed three times in dH_2O and processed as described above. The signal was normalized to PKD1^{KD} (0P) at 50 nM.

Autophosphorylation time course experiments were performed at 30°C with 50 nM PKD1^{KD}. Samples were taken from the reaction volume, terminated and aliquots corresponding to 0.5 pmol of PKD1^{KD} were subjected to SDS-PAGE and processed as described above.

Substrate phosphorylation time course assays with PKD1$^{ULD\text{-}5G/10G\text{-}KD}$ were performed on ice with 50 nM protein and 100 µM biotinylated Syntide2 (biotin-PLARTSVAG (GenScript)). Samples were taken from the reaction volume, terminated, and spotted on a nitrocellulose membrane. The membrane was washed five times with 20 ml of 75 mM H_3PO_4 and exposed to a phosphor screen for 2 h together with a set of internal standards.

Michaelis-Menten kinetics were performed on ice with 0-150 µM biotin-Syntide 2. The reactions were started by adding 10 nM PKD1^{KD} and terminated after 2 min. Aliquots were spotted onto a nitrocellulose membrane and processed as described above.
Substrate phosphorylation assays with PKD1^{KD} (D665N) were done with 50 nM PKD1^{KD} (D665N) and 0-100 µM Syntide 2 (Sigma).

For the analysis of PKD1^{KD} autophosphorylation by intact mass spectrometry 20 mL of kinase reaction (20 mM HEPES pH 7.4, 150 mM KCl, 1 mM TCEP, 1 mM EDTA, 1 % glycerol, 0.25 % CHAPS, 5 mM $MgCl_2$ 1 mM ATP, 10 nM short PKD1^{KD}, 40 nM PKD1^{KD} D706N) were incubated at 30°C for 7 h. The reaction was terminated by the addition of 10 mM EDTA and the solution was concentrated to 70 µL in a 10 kDa cutoff Vivaspin concentrator (Sartorius) and the protein was buffer exchanged with 20 mM HEPES pH 7.4, 150 mM KCl, 1 mM TCEP, 1 mM EDTA, 1 % glycerol in a Zeba desalting column (Thermo Fisher Scientific). An unphosphorylated reference sample was prepared with 2 µM short PKD1^{KD} and 8 µM PKD1^{KD} D706N in the same buffer. Sample preparation for the autophosphorylation analysis of GST-tagged PKD^{KD} proteins by intact mass spectrometry was carried out analogously, with the exception of an additional TEV cleavage step at 4°C before concentration and buffer exchange.

Dynamic light scattering

Dynamic light scattering measurements were performed on 10 µM PKD^{KD} in 100 mM Tris pH 7.5, 150 mM KCl, 1 mM TCEP, 1% Glycerol, 10 mM $MgCl_2$ 1 mM ATP at 20°C using a DynaPro NanoStar instrument (Wyatt Technology Corp.). Buffer and protein stocks were cleared from aggregate particles by ultracentrifugation at 186,000 x g for 1.5 h prior to the measurements.

HDX-MS

Protein digestion and MS/MS data collection

Protein samples were rapidly thawed and injected onto an integrated fluidics system containing a HDx-3 PAL liquid handling robot and climate-controlled (2°C) chromatography system (LEAP Technologies), a Dionex Ultimate 3000 UHPLC system, as well as an Impact HD QTOF Mass spectrometer (Bruker). The full details of the automated LC system are described in (9). The protein was run over one immobilized pepsin column (Trajan; ProDx protease column, 2.1 mm x 30 mm PDX.PP01-F32) at 200 µL/min for 3 minutes at 8°C. The resulting peptides were collected and desalted on a C18 trap column (Acquity UPLC BEH C18 1.7mm column (2.1 x 5 mm); Waters 186003975). The trap was subsequently eluted in line with an ACQUITY 1.7 µm particle, 100 × 1 mm^2 C18 UPLC column (Waters), using a gradient of 3-35% B (Buffer A 0.1% formic acid; Buffer B 100% acetonitrile) over 11 minutes immediately followed by a gradient of 35-80% over 5 minutes. Mass spectrometry experiments acquired over a mass range from 150 to 2200 m/z using an electrospray ionization source operated at a temperature of 200°C and a spray voltage of 4.5 kV.

Peptide identification

Peptides were identified from the non-deuterated samples of $PKD1^{KD}$ or $PKD1^{KD}$ S738/742E using data-dependent acquisition following tandem MS/MS experiments (0.5 s precursor scan from 150-2000 m/z; twelve 0.25 s fragment scans from 150-2000 m/z). MS/MS datasets were analyzed using PEAKS7 (PEAKS), and peptide identification was carried out by using a false discovery-based approach, with a threshold set to 0.1% using a database of known contaminants found in Sf9 cells (10). The search parameters were set with a precursor tolerance of 20 ppm, fragment mass error 0.02 Da, charge states from

1-8, leading to a selection criterion of peptides that had a -10logP score of 26.2 and 24.8 for PKD1^{KD} or PKD1^{KD} S738/742E, respectively.

Mass Analysis of Peptide Centroids and Measurement of Deuterium Incorporation

HD-Examiner Software (Sierra Analytics) was used to calculate the level of deuterium incorporation into each peptide. All peptides were manually inspected for correct charge state, correct retention time, and appropriate selection of isotopic distribution. Deuteration levels were calculated using the centroid of the experimental isotope clusters. Results are presented as relative levels of deuterium incorporation, with no correction for back exchange. The only correction was for the deuterium percentage of the buffer in the exchange reaction (71.3%). Differences in exchange in a peptide were considered significant if they met all three of the following criteria: ≥5% change in exchange, ≥0.5 Da difference in exchange, and a 2-tailed T-test value of less than 0.01 at any time point. The raw HDX data are shown in two different formats. The raw peptide deuterium incorporation graphs for a selection of peptides with significant differences are shown in Supplementary Figure S5A, with the raw data for all analyzed peptides in the source data. To allow for visualization of differences across all peptides, we utilized number of deuteron difference (#D) plots (Figure 3B). These plots show the total difference in deuterium incorporation over the entire H/D exchange time course, with each point indicating a single peptide. Samples were only compared within a single experiment and were never compared to experiments completed at a different time with a different final D_2O level. The data analysis statistics for all HDX-MS experiments are in Supplemental Table 2 according to published guidelines (11). The mass spectrometry proteomics data have been deposited to the ProteomeXchange Consortium via the PRIDE partner repository (8) with the dataset identifier PXD033139.

Protein extraction of cells and Western blotting

Whole cell extracts were obtained by solubilizing cells in lysis buffer (20 mM Tris pH 7.4, 150 mM NaCl, 1% Triton X-100, 1 mM EDTA, 1 mM ethylene glycol tetra acetic acid (EGTA), plus Complete protease inhibitors and PhosSTOP (Roche Diagnostics, Basel, Switzerland)). Whole cell lysates were clarified by centrifugation for 15 min at 16,000 g and 4°C. Equal amounts of protein were loaded on 10% polyacrylamide gels or were run on NuPage Novex 4–12% Bis-Tris gels (Life Technologies) and blotted onto nitrocellulose

membranes using the iBlot device (Life Technologies). Membranes were blocked for 30 min with 0.5% (v/v) blocking reagent (Roche Diagnostics) in PBS containing 0.05% (v/v) Tween-20. Membranes were incubated with primary antibodies overnight at 4°C, followed by 1 hr incubation with HRP-conjugated or IRDye-conjugated secondary antibodies at room temperature.

Proteins were visualized using an enhanced chemiluminescence detection system (Thermo Fisher Scientific, Waltham, MA, USA) or the Licor Odyssey system. Special care was taken not to overexpose in order to guarantee accurate quantifications. Densitometry was performed using Image Studio Lite 4.0 (Li-COR Biosciences, Bad Homburg, Germany). For each protein, the integrated density of the signal was measured and corrected for background signals.

Immunofluorescence staining and confocal microscopy

Cells expressing PKD1-EGFP were grown on glass coverslips coated with 2,5 mg/ml collagen R (Serva, Heidelberg, Germany) and fixed for 15 min with 4% (v/v) paraformaldehyde. After washes in PBS, cells were incubated for 5 min with 1 M glycine in PBS and permeabilized for 2 min with 0.1% (v/v) Triton X-100 in PBS. Blocking was performed with 5% (v/v) bovine serum (PAN) in PBS for 30 min. Fixed cells were incubated with primary antibodies diluted in blocking buffer for 2 hr at room temperature. Following three washing steps with PBS, cells were incubated with Alexa-Fluor-546-labeled secondary antibodies in blocking buffer for 1 hr at room temperature. Nuclei were counterstained with DAPI and mounted in ProLong Gold Antifade Reagent (Thermo Fisher Scientific). All samples were analyzed at room temperature using a confocal laser scanning microscope (LSM 710, Carl Zeiss) equipped with a Plan Apochromat 63x/1.40 DIC M27 (Carl Zeiss, Jena, Germany) oil-immersion objective. GFP was excited with the 488 nm line of an Argon laser, its emission was detected from 496 to 553 nm. Alexa546 was excited with a 561 nm DPSS laser, its emission was detected from 566 to 622 nm. Image acquisition for G-PKDrep ratiometric imaging was done as follows: z-stacks of 0.5 mm intervals were acquired throughout the cell and maximum intensity projections were calculated. GFP and Alexa546 channels were hereby acquired with the same pinhole setting that was adjusted to 1 AU in the Alexa 546 channel. Laser powers were adjusted to prevent fluorophore saturation and identical photomultiplier tube and laser settings were maintained throughout the whole experiment. Image

processing and analysis was performed with Zen black 2.1 software. Regions of interest of identical Golgi areas of reporter expressing cells were selected in the GFP channel, mean pixel intensity values of the selected areas in both channels were measured and the Alexa546 to GFP ratio was calculated.

In-cell membrane translocation assay

For transfection into mammalian cells, wild type and mutant constructs of full-length PKD1 were cloned into the EGFP-C1 and mCherry-C1 vectors (Clontech), resulting in N-terminally labelled fusion proteins. With the exception of $PKD1^{K612N}$, which was expressed as an EGFP fusion, all mutant PKD1 variants were expressed as mCherry fusions.

COS7 cells were seeded into a 4-well chamber microscopy dish (InVitro Scientific) and co-transfected with wild type and mutant constructs of PKD 20 h post-seeding with 250 ng of wild-type and 250 ng mutant plasmid DNA per well using TurboFect (Thermo Scientific) according to the manufacturer's instructions. After 24 h, the medium was exchanged to Hank's Buffered Saline Solution (Life Technologies), and cells were imaged in HBSS on a Zeiss LSM710 confocal microscope equipped with 488- and 561-nm lasers. Membrane translocation in live cells was induced by the addition of 500 nM phorbol 12-myristate 13-acetate (PMA; Sigma) and monitored by imaging in 20–30 s intervals. The decay of cytosolic fluorescence over time was quantified in a region of interest using ImageJ, and the half-maximum translocation time ($t_{0.5}$) was determined by a logistic fit function over the decay curve. Mutations were considered to impact membrane translocation if the ratio of $t_{0.5}$ for the wild type over the mutant protein deviated significantly from 1. For visualization, the data curves were normalized to the minimum and maximum values of fluorescence in the cell.

Supplementary References

1. Y. F. Fuchs, *et al.*, A Golgi PKD activity reporter reveals a crucial role of PKD in nocodazole-induced Golgi dispersal. *Traffic (Copenhagen, Denmark)* **10**, 858–67 (2009).
2. A. Hausser, *et al.*, Structural requirements for localization and activation of protein kinase C µ (PKCµ) at the Golgi compartment. *Journal of Cell Biology* **156**, 65–74 (2002).
3. D. J. Elsner, K. M. Siess, T. Gossenreiter, M. Hartl, T. A. Leonard, A ubiquitin-like domain controls protein kinase D dimerization and activation by trans-autophosphorylation. *The Journal of biological chemistry* **294**, 14422–14441 (2019).
4. J. Rappsilber, M. Mann, Y. Ishihama, Protocol for micro-purification, enrichment, pre-fractionation and storage of peptides for proteomics using StageTips. *Nature protocols* **2**, 1896–906 (2007).
5. S. Tyanova, T. Temu, J. Cox, The MaxQuant computational platform for mass spectrometry-based shotgun proteomics. *Nature Protocols* **11**, 2301–2319 (2016).
6. Z. L. Chen, *et al.*, A high-speed search engine pLink 2 with systematic evaluation for proteome-scale identification of cross-linked peptides. *Nature Communications 2019 10:1* **10**, 1–12 (2019).
7. C. W. Combe, L. Fischer, J. Rappsilber, xiNET: Cross-link Network Maps With Residue Resolution. *Molecular & Cellular Proteomics* **14**, 1137–1147 (2015).
8. Y. Perez-Riverol, *et al.*, The PRIDE database resources in 2022: a hub for mass spectrometry-based proteomics evidences. *Nucleic acids research* **50**, D543–D552 (2022).
9. J. T. B. Stariha, R. M. Hoffmann, D. J. Hamelin, J. E. Burke, Probing Protein-Membrane Interactions and Dynamics Using Hydrogen-Deuterium Exchange Mass Spectrometry (HDX-MS). *Methods in molecular biology (Clifton, N.J.)* **2263**, 465–485 (2021).
10. J. M. Dobbs, M. L. Jenkins, J. E. Burke, Escherichia coli and Sf9 Contaminant Databases to Increase Efficiency of Tandem Mass Spectrometry Peptide Identification in Structural Mass Spectrometry Experiments. *Journal of the American Society for Mass Spectrometry* **31**, 2202–2209 (2020).
11. G. R. Masson, *et al.*, Recommendations for performing, interpreting and reporting hydrogen deuterium exchange mass spectrometry (HDX-MS) experiments. *Nature methods* **16**, 595–602 (2019).

Figure S1. Mass spectra of all protein constructs employed in this study.

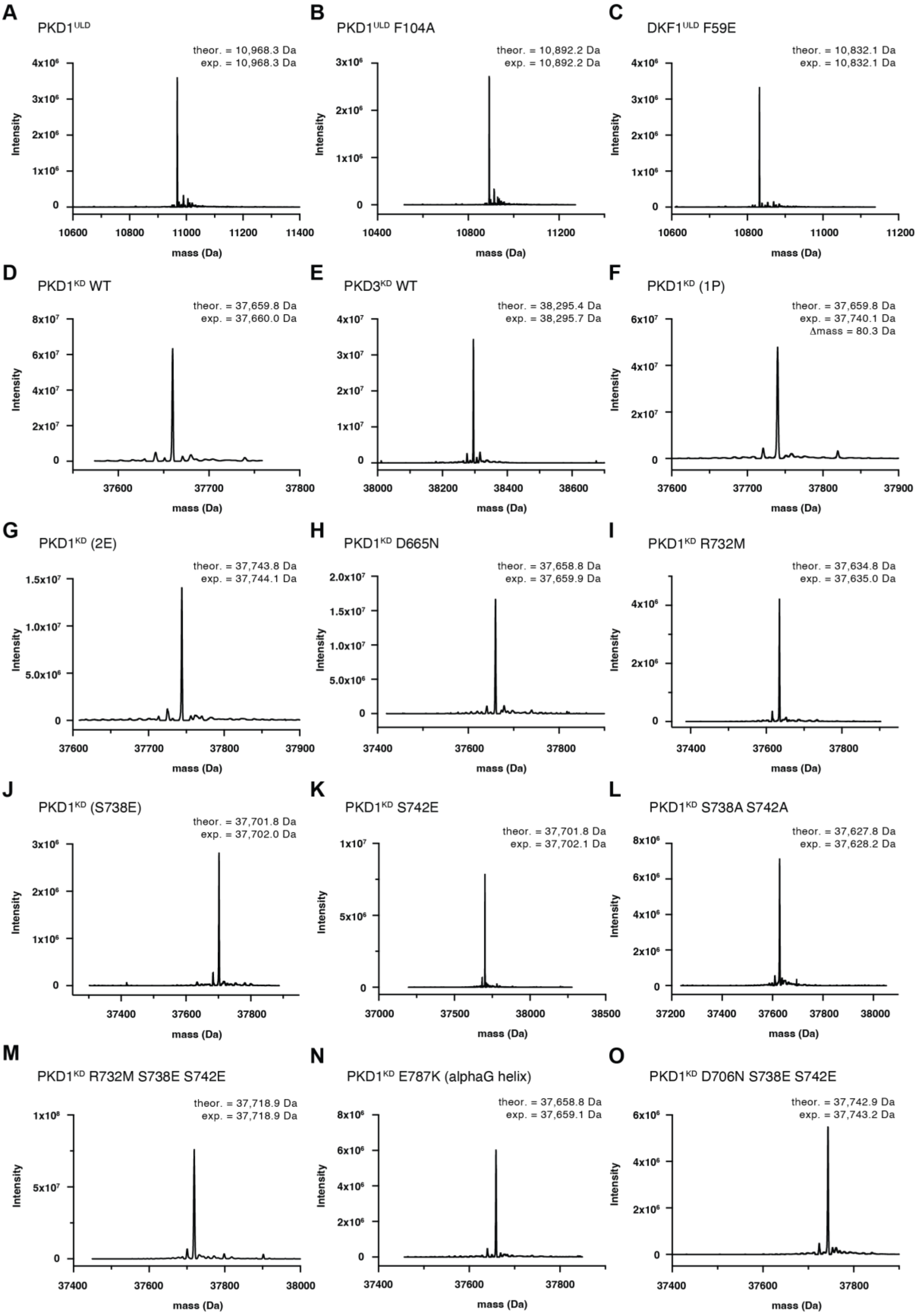

Figure S2. Mass spectra of all protein constructs employed in this study.

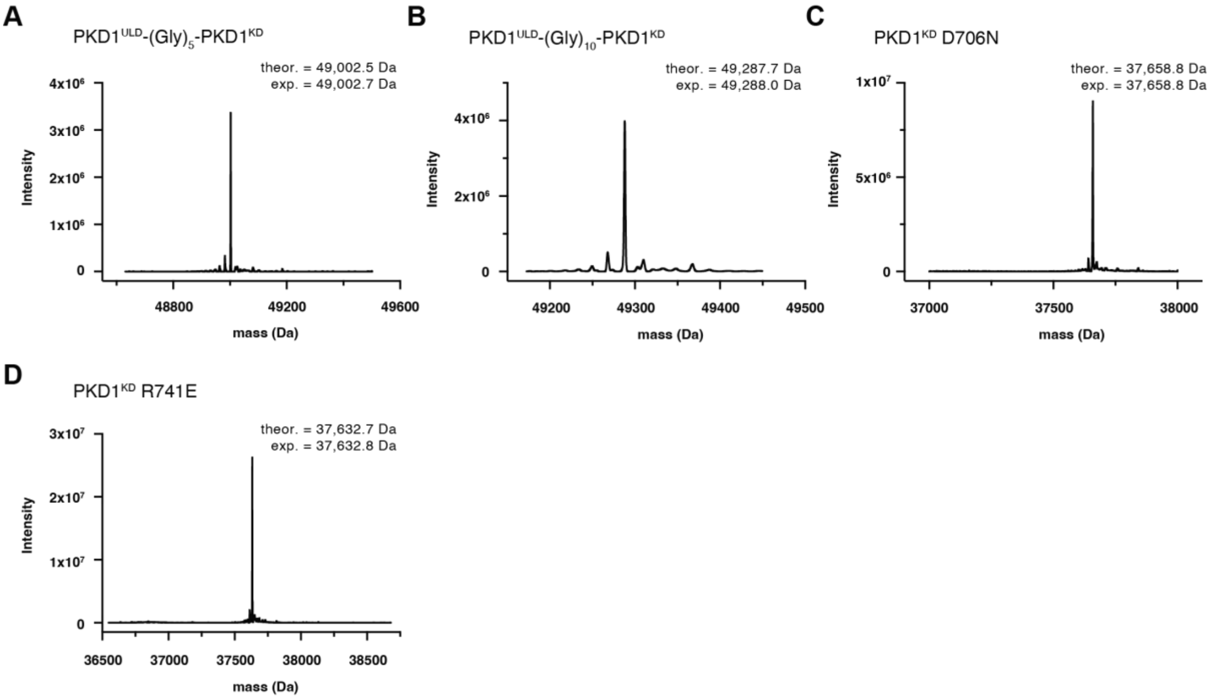

Figure S3. The PKD kinase domain forms a face-to-face dimer.

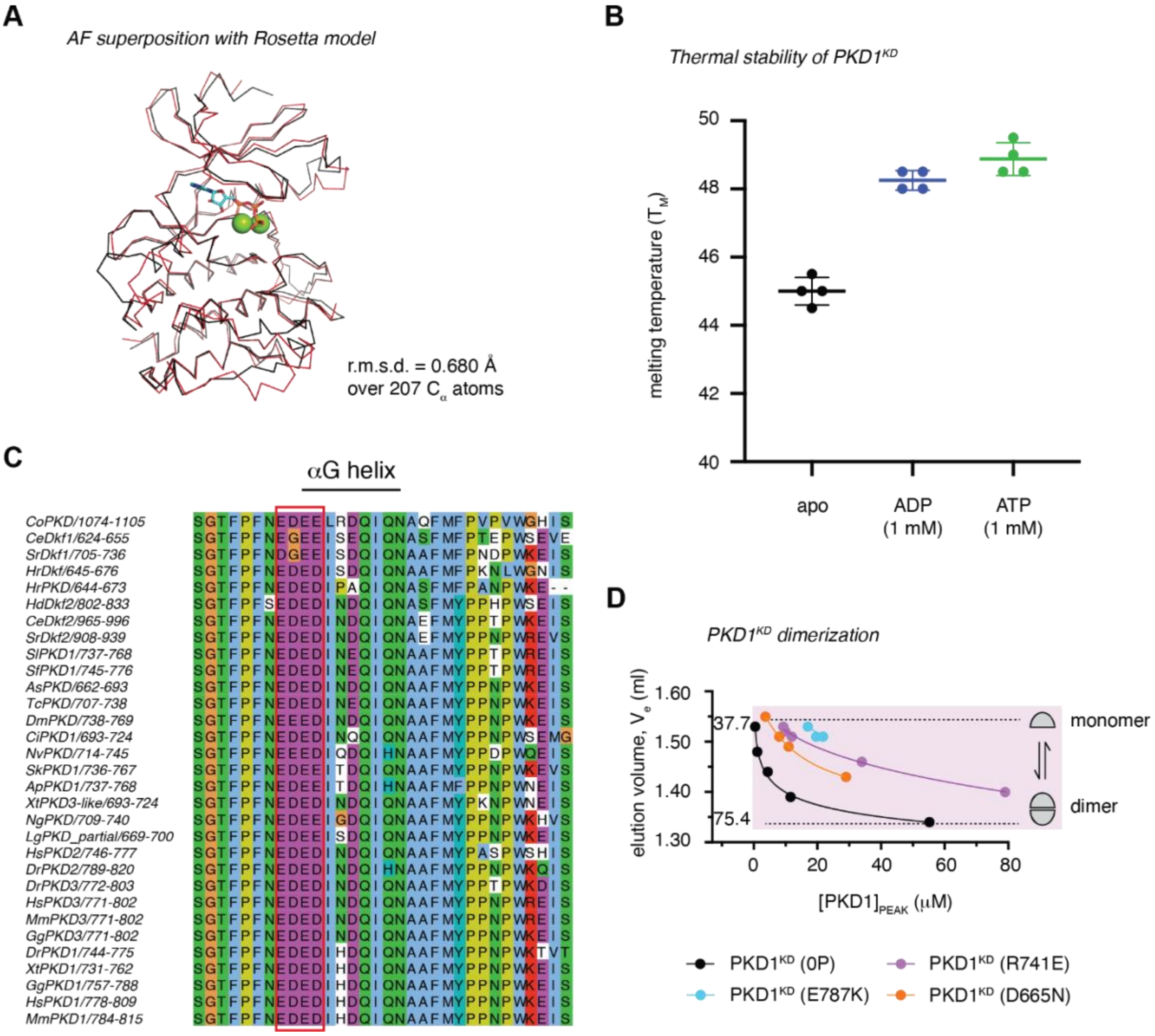

A. Superposition of *in silico* models of PKD1 kinase domain derived from Rosetta and AlphaFold.

B. Thermal stability of PKD1^{KD} in the absence (black) or presence of 1 mM ADP (blue) or ATP (green).

C. Sequence conservation of the αG helix of PKD orthologs.

D. Analytical size-exclusion chromatography of PKD1^{KD} variants. Dashed lines correspond to monomer and dimer, calibrated by multi-angle light scattering to determine absolute molecular mass.

Figure S4. Dimerization of the PKD kinase domain is autoinhibitory.

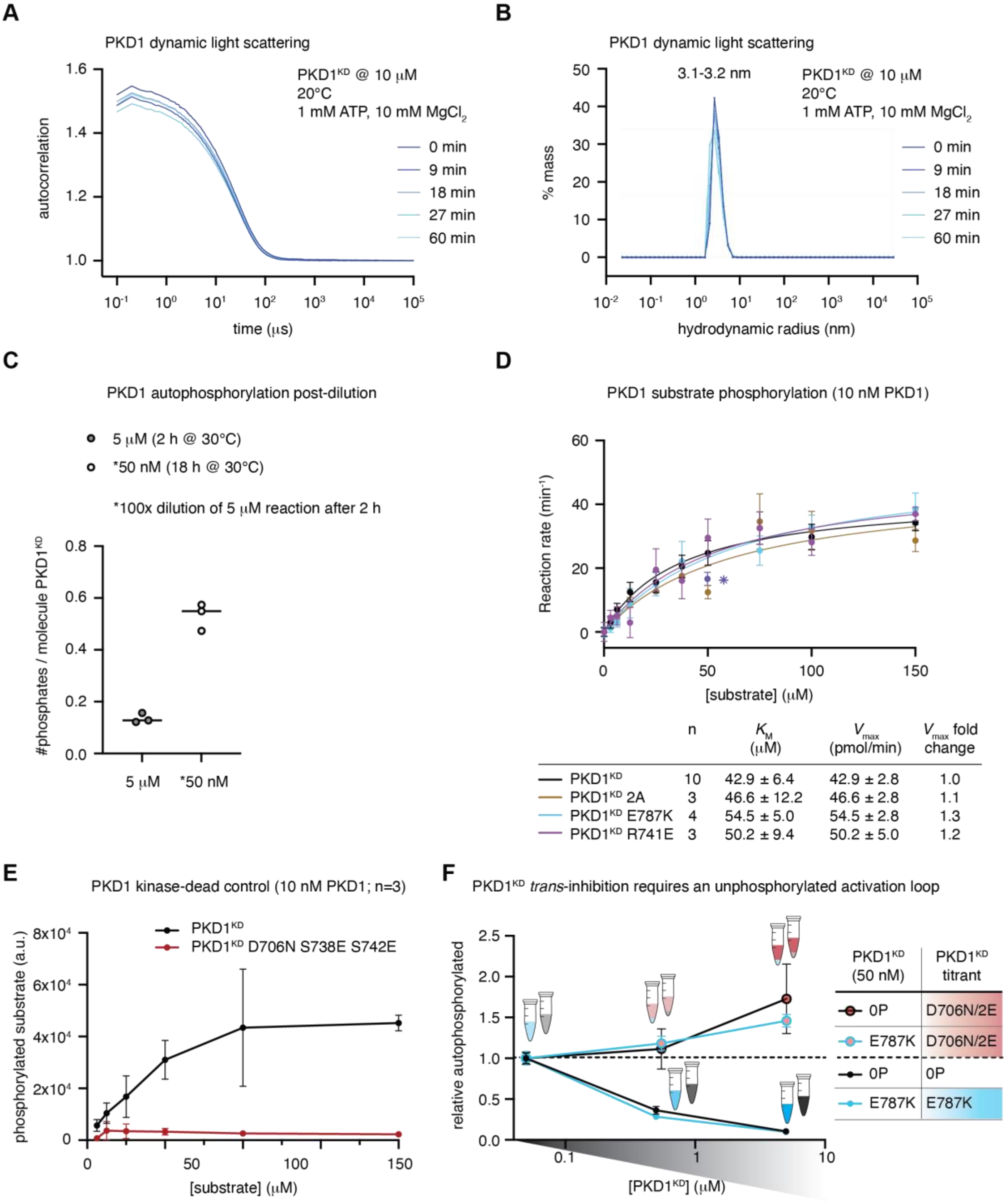

A. Dynamic light scattering autocorrelation curves of PKD1^{KD} at 10 μM during the course of a 1 h autophosphorylation assay.

B. Hydrodynamic radius of PKD1^{KD} particles calculated from the autocorrelation curves in A.

C. Recovery of PKD1 autophosphorylation activity following dilution. A 5 μM reaction that had reached completion after 2 h was diluted to 50 nM in reaction buffer and incubated for a further 18 h. The stoichiometry of phosphorylation is plotted for each.

D. Substrate phosphorylation kinetics of PKD1^{KD} E787K and R741E. Error bars are the standard deviation of *n* biologically independent experiments. Table indicates the number (*n*) of independent biological replicates and the values for K_M and V_{max} derived from fitting the data with the Michaelis-Menten equation. Asterisk denotes removal of one outlier.

E. Substrate phosphorylation kinetics of PKD1^{KD} D706N S738E S742E. Error bars are the standard deviation of 3 biologically independent experiments.

F. Autophosphorylation of wild-type PKD1^{KD} and PKD1^{KD} E787K in the presence or absence of catalytically dead, dimerization-incompetent PKD1^{KD} D706N/2E. Error bars are the standard deviation of 3 biologically independent experiments.

Figure S5. Dimerization of the PKD kinase domain is autoinhibitory.

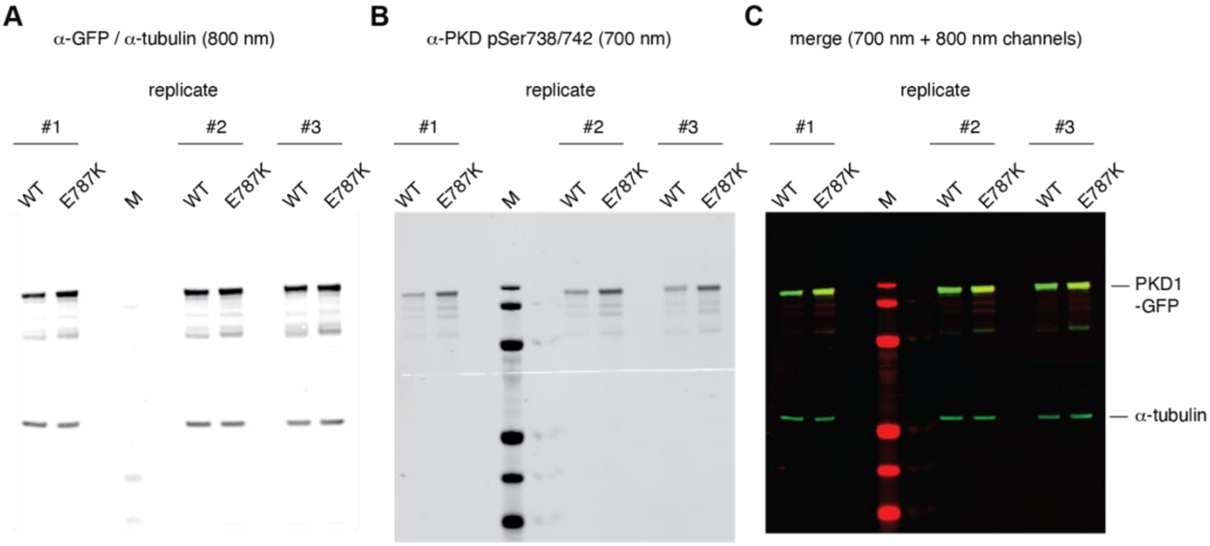

A. Western blots quantified in Figure 2D. Blot probed with anti-GFP and anti-tubulin primary antibodies and measured at 800 nm.

B. Western blots quantified in Figure 2D. Blot probed with anti-PKD1 pS738/pS742 primary antibody and measured at 700 nm.

C. Merged image of the two channels shown in panels A and B.

Figure S6. PKD1 activation loop autophosphorylation occurs in *cis*.

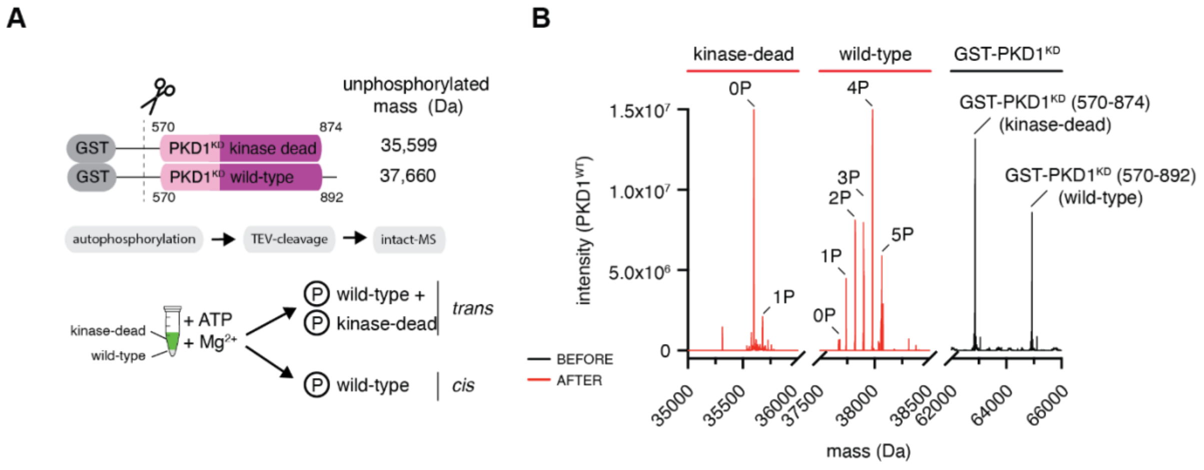

A. Cartoon schematic of the autophosphorylation reaction containing 10 nM wild-type GST-PKD1^{KD} (570-892) and 40 nM kinase-dead GST-PKD1^{KD} D706N (570-874).

B. Mass spectrometry analysis of PKD1^{KD} following autophosphorylation and TEV cleavage, as depicted in A. Phospho-species of wild-type PKD1^{KD} are separated by 80 Da.

Figure S7. Activation loop autophosphorylation increases PKD1 catalytic activity.

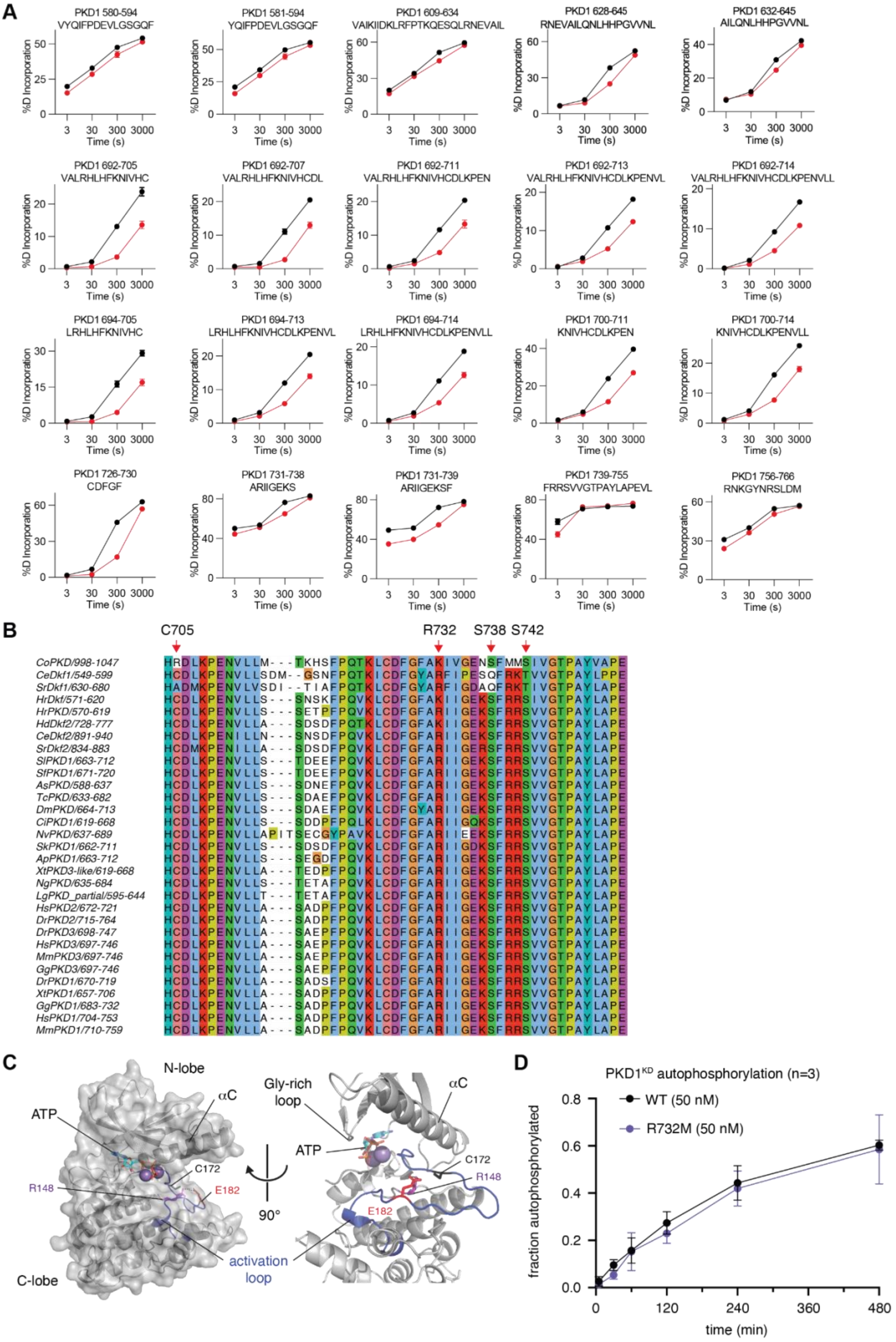

A. Deuterium incorporation for selected PKD1^{KD} peptides. Error bars are the standard deviation of 3 biologically independent experiments.
B. Sequence conservation in the catalytic and activation loops of PKD orthologs. Key residues in activation loop phosphate coordination are indicated with red arrows.
C. Coordination of the activation loop (blue cartoon) in phosphorylase kinase. E182 (red sticks) occupies the position of the canonical phosphoserine in PKD1 and is coordinated by R148 (purple sticks) in the HRD motif.
D. Autophosphorylation kinetics of wild-type PKD1^{KD} compared to PKD1^{KD} R732M. Error bars are the standard deviation of 3 biologically independent experiments.

Figure S8. Activation loop autophosphorylation increases PKD1 catalytic activity.

A

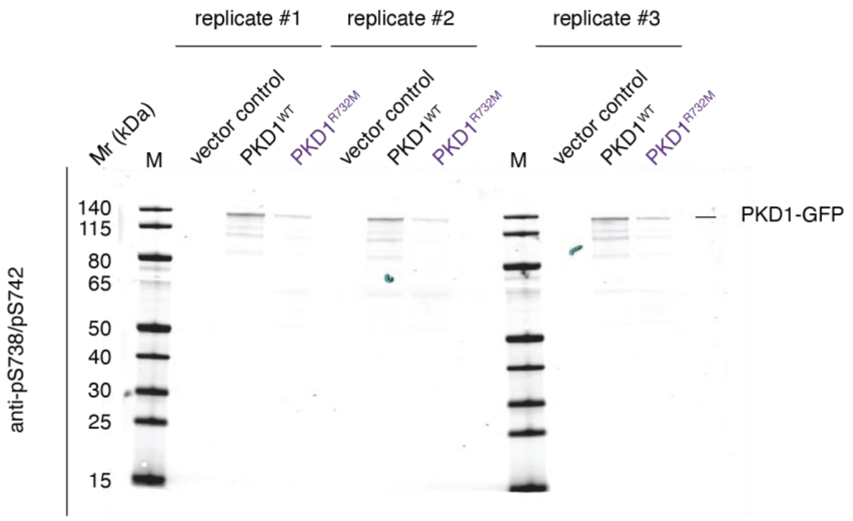

B

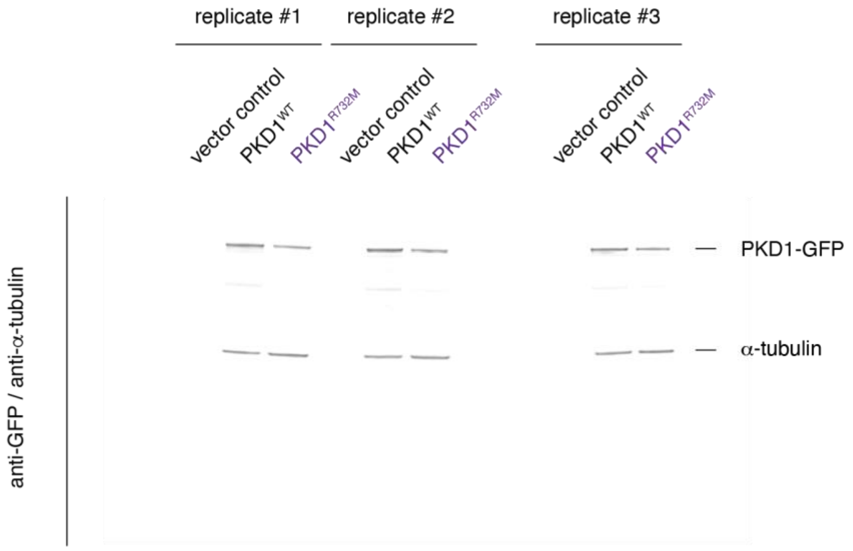

A. Western blots quantified in Figure 4F. Blot probed with anti-PKD1 pS738/pS742 antibody.

B. Western blots quantified in Figure 4F. Blots probed with anti-GFP and anti-α-tubulin antibodies.

Figure S9. PKD auto- and substrate phosphorylation are mechanistically distinct.

A

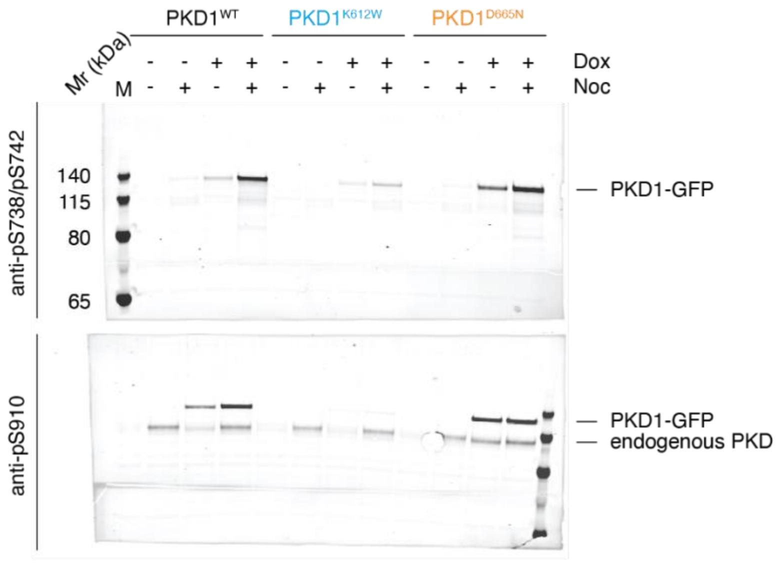

B

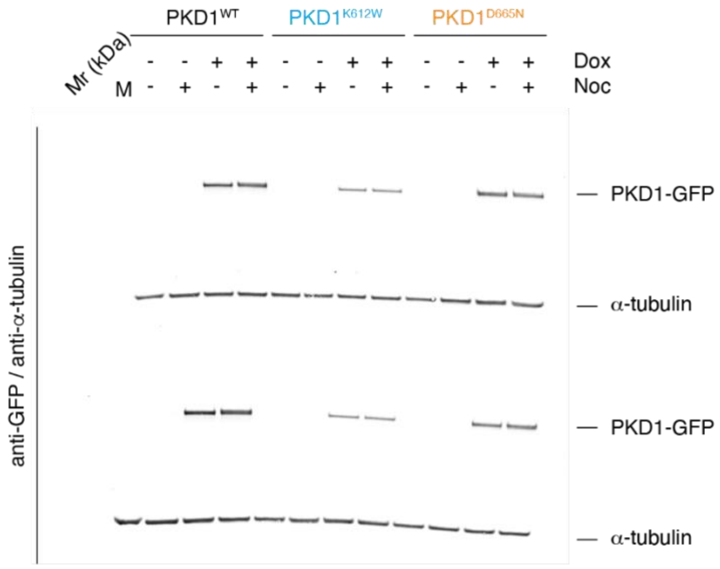

A. Western blots quantified in Figure 5E-F. Upper blot probed with anti-PKD1 pS738/pS742 antibody. Lower blot probed with anti-PKD1 pS910 antibody.
B. Western blots quantified in Figure 5E-F. Blot probed with anti-GFP and anti-α-tubulin antibodies.

Figure S10. Substrate binding-deficient and constitutively dimeric PKD1 block secretion.

A

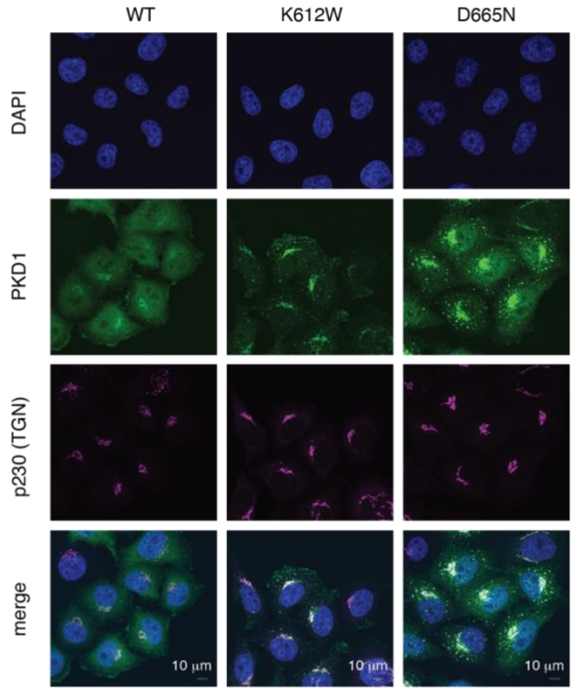

A. Subcellular localization of wild-type PKD1-GFP, PKD1^{K612W}-GFP (ATP binding mutant) and PKD1^{D665N}-GFP (substrate binding mutant) in stable Flp-In TRex HeLa cells.

Figure S11. Substrate binding-deficient and constitutively dimeric PKD1 block secretion.

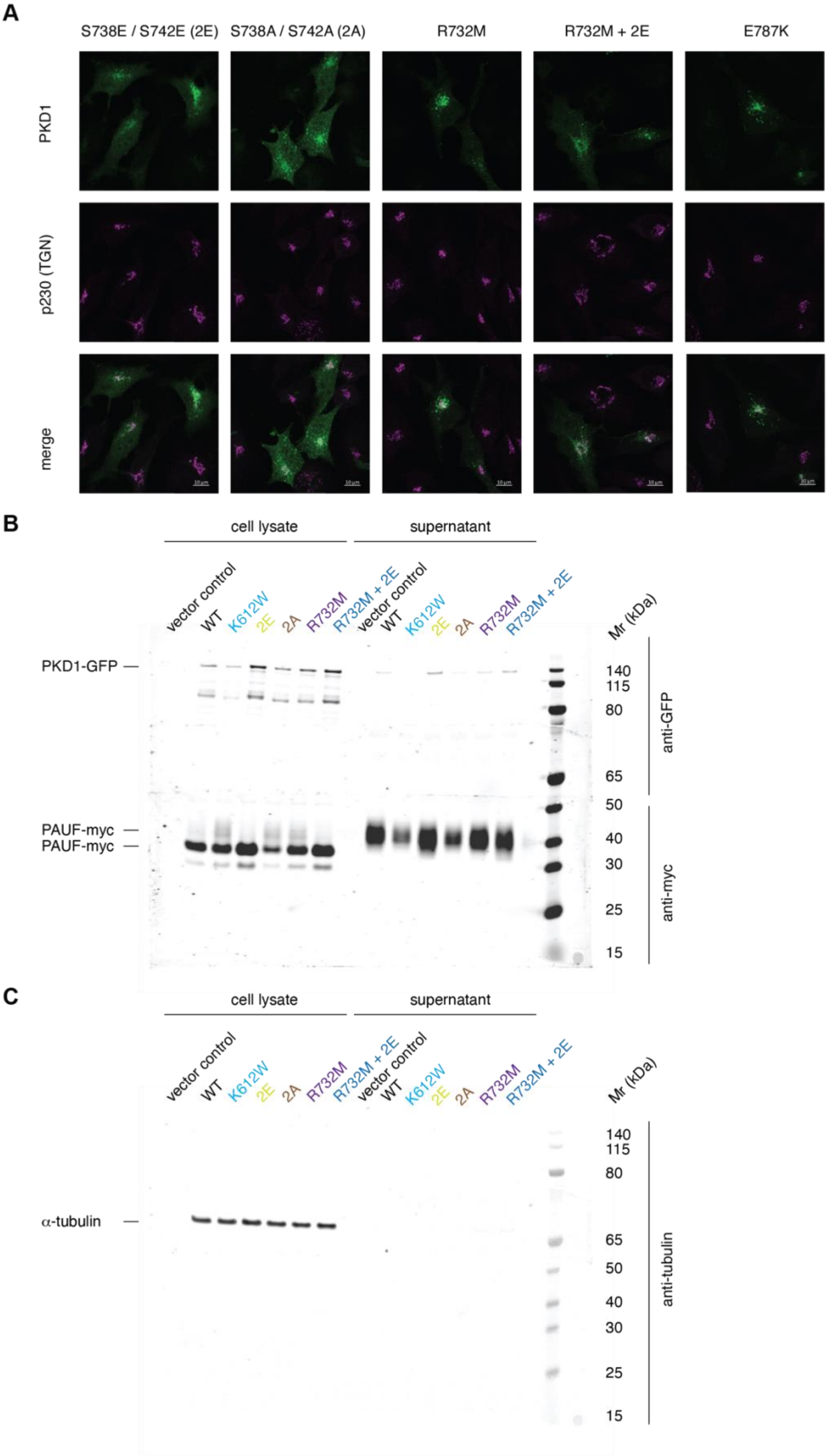

A. Co-localization of PKD1 mutants with a TGN marker (p230) in stable Flp-In TRex HeLa cells.
B. Western blot quantified in Figure 6C. Upper blot probed with anti-GFP antibody. Lower blot probed with anti-myc antibody.
C. Western blot loading control for Figure 6C. Blot probed with anti-α-tubulin antibody.

Figure S12. Autoregulation of membrane binding depends on ATP, but not dimerization.

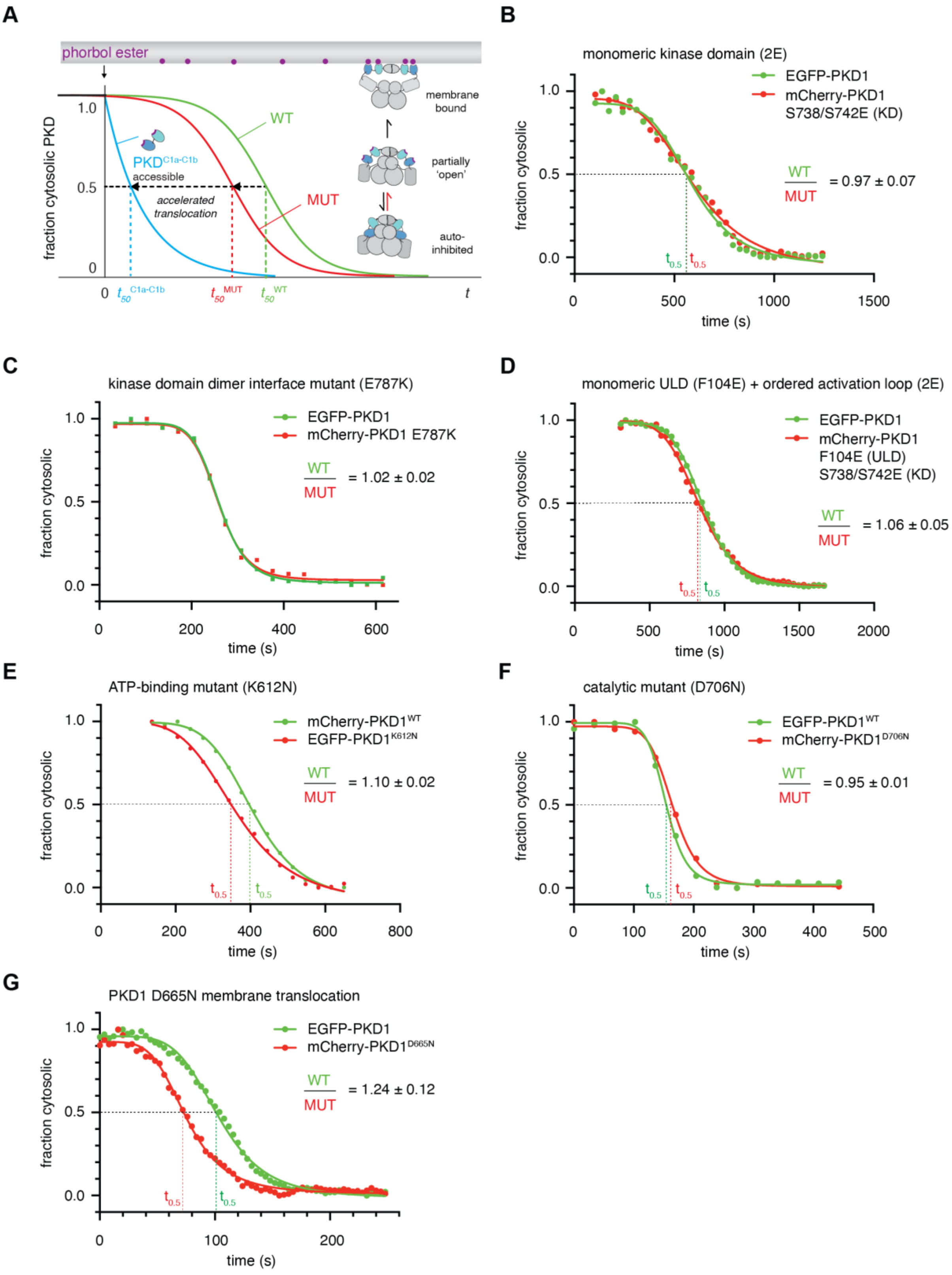

A. Cartoon schematic of PKD membrane translocation assay. The tandem DAG-binding domains (blue) translocate rapidly to the plasma membrane upon phorbol ester treatment. Mutations that destabilize the intramolecular assembly

of PKD accelerate its membrane translocation kinetics (red) compared to wild-type PKD (green).

B. Membrane translocation kinetics of PKD1^{2E} (mCherry, red) compared to wild-type PKD1 (EGFP, green) in Cos7 cells. Ratio of times for half-maximal translocation derived from analysis of 4 cells.
C. Membrane translocation kinetics of PKD1^{E787K} (mCherry, red) compared to wild-type PKD1 (EGFP, green) in Cos7 cells. Ratio of times for half-maximal translocation derived from analysis of 4 cells.
D. Membrane translocation kinetics of PKD1$^{F104E+2E}$ (mCherry, red) compared to wild-type PKD1 (EGFP, green) in Cos7 cells. Ratio of times for half-maximal translocation derived from analysis of 4 cells.
E. Membrane translocation kinetics of PKD1^{K612N} (EGFP, red) compared to wild-type PKD1 (mCherry, green) in Cos7 cells. Ratio of times for half-maximal translocation derived from analysis of 3 cells.
F. Membrane translocation kinetics of PKD1^{D706N} (mCherry, red) compared to wild-type PKD1 (EGFP, green) in Cos7 cells. Ratio of times for half-maximal translocation derived from analysis of 5 cells.
G. Membrane translocation kinetics of wild-type EGFP-PKD1 (green) compared to mCherry-PKD1^{D665N} (red) in Cos7 cells. Ratio of times for half-maximal translocation derived from analysis of 3 cells.

Supplementary Table S1. EDC cross-linked peptides (PDK1^{KD}).

Crosslinked residues		Crosslinked peptides		Abundance Mono-mer	Abundance Di-mer	Abundance Total
736	622	IIGEK(4)	FPTKQESQLR(4)	7	3	10
615	622	IIDK(3)	FPTKQESQLR(4)	1	2*	3
736	758	IIGEK(4)	NKGYNR(2)	6	2	8
737	668	IIGEKSFR(5)	LHGDMLEMILSSEK(7)	4	0	4
616	624	IIDKLR(4)	QESQLR(2)	5	5*	10
737	624	IIGEKSFR(5)	QESQLR(2)	1	0	1
736	737	IIGEKSFRR(4)(5)		6	0	6
708	710	NIVHCDLKPENVLLASADPFPQVK(8)(10)		7	2	9
852	854	ELECKIGER(3)(5)		2	0	2
622	624	FPTKQESQLR(4)(6)		18	0	18
674	675	LHGDMLEMILSSEKGR(13)(14)		1	0	1
612	615	DVAIKIIDK(5)(8)		1	0	1
850	854	ELECKIGER(1)(5)		2	0	2
831	622	YSVDK(4)	FPTKQESQLR(4)	0	2	2
668	622	LHGDMLEMILSSEK(7)	FPTKQESQLR(4)	0	4*	4
708	718	NIVHCDLKPENVLLASADPFPQVK(8)(18)		0	3	3
706	708	NIVHCDLKPENVLLASADPFPQVK(6)(8)		0	1	1

*cross-links used as experimental restraints in Rosetta

Supplementary Table S2. HDX Data Summary

Protein Data Set	PKD1cat WT/Mutant
HDX reaction details	%D2O=71.3%<br>pH(read)= 7.5<br>Temp= 18°C
HDX time course	3s, 30s, 300s, 3000s
HDX controls	N/A
Back-exchange	Corrected based on %D2O
Number of peptides	128
Sequence coverage	97.9%
Average peptide length / Redundancy	Length = 13.3<br>Redundancy = 4.9
Replicates	3
Repeatability	Average StDev = 6.0%
Significant differences in HDX	>5% and >0.5 Da and unpaired t-test <0.01
